# Synergistic Lethality of a Binary Inhibitor of Mycobacterium tuberculosis KasA

**DOI:** 10.1128/mBio.02101-17

**Published:** 2018-12-18

**Authors:** Pradeep Kumar, Glenn C. Capodagli, Divya Awasthi, Riju Shrestha, Karishma Maharaja, Paridhi Sukheja, Shao-Gang Li, Daigo Inoyama, Matthew Zimmerman, Hsin Pin Ho Liang, Jansy Sarathy, Marizel Mina, George Rasic, Riccardo Russo, Alexander L. Perryman, Todd Richmann, Aditi Gupta, Eric Singleton, Sheetal Verma, Seema Husain, Patricia Soteropoulos, Zhe Wang, Roxanne Morris, Gene Porter, Gautam Agnihotri, Padmini Salgame, Sean Ekins, Kyu Y. Rhee, Nancy Connell, Véronique Dartois, Matthew B. Neiditch, Joel S. Freundlich, David Alland

**Affiliations:** a Division of Infectious Disease, Department of Medicine, and the Ruy V. Lourenco Center for the Study of Emerging and Reemerging Pathogens, New Jersey Medical School, Rutgers, The State University of New Jersey, Newark, New Jersey, USA; b Department of Microbiology, Biochemistry and Molecular Genetics, New Jersey Medical School, Rutgers, The State University of New Jersey, Newark, USA; c Department of Pharmacology, Physiology & Neuroscience, New Jersey Medical School, Rutgers, The State University of New Jersey, Newark, New Jersey, USA; d Public Health Research Institute, New Jersey Medical School, Rutgers, The State University of New Jersey, Newark, New Jersey, USA; e Genomics Center, New Jersey Medical School, Rutgers, The State University of New Jersey, Newark, New Jersey, USA; f Division of Infectious Diseases, Department of Medicine, Weill Cornell Medical College, New York, New York, USA; g WuXi AppTec, Plainsboro, New Jersey, USA; h Collaborations Pharmaceuticals, Inc., Raleigh, North Carolina, USA; MedImmune

**Keywords:** antitubercular, DG167, KasA, *Mycobacterium tuberculosis*, mycolic acid biosynthesis, drug development, isoniazid, synergistic lethality

## Abstract

We report GSK3011724A (DG167) as a binary inhibitor of β-ketoacyl-ACP synthase (KasA) in Mycobacterium tuberculosis. Genetic and biochemical studies established KasA as the primary target. The X-ray crystal structure of the KasA-DG167 complex refined to 2.0-Å resolution revealed two interacting DG167 molecules occupying nonidentical sites in the substrate-binding channel of KasA. The binding affinities of KasA to DG167 and its analog, 5g, which binds only once in the substrate-binding channel, were determined, along with the KasA-5g X-ray crystal structure. DG167 strongly augmented the *in vitro* activity of isoniazid (INH), leading to synergistic lethality, and also synergized in an acute mouse model of M. tuberculosis infection. Synergistic lethality correlated with a unique transcriptional signature, including upregulation of oxidoreductases and downregulation of molecular chaperones. The lead structure-activity relationships (SAR), pharmacokinetic profile, and detailed interactions with the KasA protein that we describe may be applied to evolve a next-generation therapeutic strategy for tuberculosis (TB).

## Abstract (precis)

Cell wall biosynthesis inhibitors have proven highly effective for treating tuberculosis (TB). We discovered and validated members of the indazole sulfonamide class of small molecules as inhibitors of Mycobacterium tuberculosis KasA—a key component for biosynthesis of the mycolic acid layer of the bacterium’s cell wall and the same pathway as that inhibited by the first-line antitubercular drug isoniazid (INH). One lead compound, DG167, demonstrated synergistic lethality in combination with INH and a transcriptional pattern consistent with bactericidality and loss of persisters. Our results also detail a novel dual-binding mechanism for this compound as well as substantial structure-activity relationships (SAR) that may help in lead optimization activities. Together, these results suggest that KasA inhibition, specifically, that shown by the DG167 series, may be developed into a potent therapy that can synergize with existing antituberculars.

## INTRODUCTION

Tuberculosis (TB) is an ongoing global health threat, made worse by an increase in the incidence of drug-resistant Mycobacterium tuberculosis infections (1). The development of new TB drugs has not kept pace with the emergence of drug resistance. Clinical resistance to even the most recently approved drugs, bedaquiline (BDQ) and delamanid, has been identified (2), prompting concerns that TB may become untreatable. TB regimens require lengthy treatment—6 months for drug-susceptible TB and >18 months for drug-resistant TB. A lengthy regimen provides ample opportunity for partial noncompliance that can lead to both treatment failure and the emergence of new drug resistance (3–5). New TB therapies are needed to both counter emerging drug resistance and to enable shortened TB treatments (6).

Renewed efforts to find new antitubercular leads have led to the discovery of thousands of whole-cell active compounds and novel chemotypes. Many of these compounds are undergoing optimization to deliver a lead for further drug development (7–9). The cell wall is well established as one of the most vulnerable subcellular components of bacterial species, including M. tuberculosis. Inhibitors of cell wall biosynthesis disrupt the outer cell envelope, causing rapid cell death, and a number of drugs that target the cell wall such as isoniazid (INH), ethambutol (EMB), ethionamide (ETH), carbapenems, and delamanid are effective at treating clinical TB. Furthermore, many of the enzymes involved in biosynthesis of the M. tuberculosis cell wall do not have close homologs in humans, suggesting that specific inhibitors of this pathway would be less toxic. We had previously described a screen for selecting cell wall-specific antituberculars using a whole-cell reporter that signaled transcriptional induction of the *iniBAC* operon and is specifically induced by cell wall inhibitors (10). This screen led to the discovery of the thiophenes as inhibitors of polyketide synthase 13 (Pks13) (11) and of DA5/DA8 as inhibitors of MmpL3 (12).

The mycobacterial cell wall is adorned with essential mycolic acids, which are synthesized by a fatty acid synthase-II (FAS-II) system that is absent in humans. The FAS-II complex includes one operon that encodes the β-ketoacyl-ACP synthases KasA and KasB and the acyl-carrier protein (AcpM) and a second operon that encodes the ketoreductase (MabA) and the enoyl reductase (InhA) (13, 14). This complex carries out cyclic elongation of short-chain fatty acids to produce long-chain meromycolic acids (C_48_ to C_64_) (15) that are condensed with C_26_ fatty acids to yield branched mycolic acids by Pks13 (11, 16). Mycolic acid variants not only are critical for pathogenesis, virulence, and persistence (15, 17, 18) but are also effective targets for antitubercular drugs. For example, INH, one of the most effective first-line antitubercular drugs, targets InhA. KasA has also been shown to be essential and a vulnerable target in mycobacteria (19). Unfortunately, the previously known inhibitors of KasA/KasB, thiolactomycin (TLM) (20–23) and platensimycin (24), have very poor whole-cell activity in M. tuberculosis (142 and 27 μM, respectively).

Recently, GSK3011724A (8) was described as a new inhibitor of KasA (25). At the time of the publication of the biological profiling of GSK3011724A, our efforts were nearly complete with respect to studies of the same molecule, identified as a strong *iniBAC* transcriptional inducer in the GlaxoSmithKline (GSK) library of whole-cell active antituberculars and renamed DG167 by us. The fact that *iniBAC* inducers such as INH are also known to strongly induce *kasA* in M. tuberculosis (26, 27) motivated us to explore DG167 with a specific focus on the INH-DG167 interaction. Here, we confirm many of the previously published findings and make significant corrections and extensions. The advances we report include the following: (i) X-ray crystallographic studies that demonstrated a unique binding of two interacting DG167 molecules to nonidentical sites in each subunit of its KasA target, (ii) potential therapeutic advantages of inhibiting FAS-II by simultaneously targeting InhA and KasA, and (iii) structure-activity relationships (SAR) around DG167. These results provide a roadmap for structure-based chemical optimization of DG167 both as a probe and an antitubercular lead.

## RESULTS

### DG167 is an inhibitor of M. tuberculosis KasA.

We screened a library of 168 compounds with established antitubercular whole-cell efficacy for putative cell wall inhibitors (8) using a previously described M. bovis BCG strain harboring a *lacZ* reporter fused to the M. tuberculosis *iniBAC* promoter (P*_iniBAC_*) (10–12). GSK3011724A (8) (renamed DG167) was found to be one of the top inducers of *iniBAC* promoter (approximately 12-fold induction), suggesting that DG167 was likely to inhibit a component of cell wall biosynthesis. DG167 possessed potent whole-cell activity against M. tuberculosis, with a MIC of 0.39 μM. Activity was preserved against both drug-susceptible and drug-resistant clinical M. tuberculosis strains and against M. tuberculosis strains resistant to MmpL3 inhibitors such as SQ109, suggesting a novel target for DG167 (Table 1). Interestingly, DG167 lacked whole-cell activity (MIC > 100 μM) against nontuberculous mycobacteria (NTM) such as M. abscessus, M. fortuitum, M. avium, M. smegmatis, and M. marinum (Table 1). We selected for DG167-resistant M. tuberculosis mutants to gain insights into its molecular target. Resistant mutants were obtained at a frequency of 1 × 10^−7^ at 8×. All seven DG167-resistant mutants analyzed (MIC shift of 4× to >256×) remained susceptible to INH, EMB, ETH, rifampin (Rif), moxifloxacin (Moxi), PA824, SQ109, and BDQ (Table 2). Whole-genome sequencing (WGS) of each mutant identified a unique single-nucleotide polymorphism (SNP) in the M. tuberculosis *kasA* gene (Table 2), supporting the earlier report (25). Computational docking studies were performed with the known KasA structure (28), and we noted that DG167 was predicted to bind the KasA substrate-binding site (see Fig. S1 in the supplemental material), in agreement with data from a cocrystal study reported by Abraham et al. (25).

**TABLE 1:** DG167 activity in laboratory and clinical strains of *Mycobacterium* species^*a*^

Strain	Type	Resistance	DG167 MIC (µM)	INH MIC (µM)
M. tuberculosis H37Rv	Laboratory	Susceptible	0.39	0.4
M. bovis BCG	Laboratory	Susceptible	0.39	0.4
M. tuberculosis DRM5 (12)	Laboratory	SQ109, DA5^*b*^	0.39	0.4
M. tuberculosis DRM8.3 (12)	Laboratory	SQ109, DA8^*b*^	0.39	0.4
M. tuberculosis mc^2^4914 (32)	Laboratory	INH	0.78	1.6
M. tuberculosis DRM12^*c*^	Laboratory	INH	0.39	0.4
M. tuberculosis 210	Clinical	Susceptible	0.39	0.4
M. tuberculosis TDR692	Clinical	Susceptible	0.39	0.2
M. tuberculosis TDR31	Clinical	INH, RIF, EMB, KAN, SM, CAP	0.2	>12
M. tuberculosis TDR36	Clinical	INH, RIF, EMB	0.39	>12
M. tuberculosis TDR116	Clinical	INH, EMB, PAS	0.2	>12
M. smegmatis	Laboratory	Wild-type	>50	>12
M. abscessus	ATCC	Wild type	>50	>12
M. avium	ATCC	Wild type	>50	>12
M. fortuitum	ATCC	Wild type	>50	>12
M. marinum	ATCC	Wild type	>50	>12

a INH, isoniazid; RIF, rifampin; EMB, ethambutol; KAN, kanamycin; SM, streptomycin; CAP, capreomycin; PAS, *para*-aminosalicylic acid.

b MmpL3 inhibitor (12).

c *M. tuberculosis* H37Rv with KatG mutation (S315T) that confers INH resistance.

**TABLE 2:** Genotypic and drug-resistance profile of DG167-resistant and KasA-overexpressing *M. tuberculosis* strains

Strain	*kasA* SNP^*a*^	KasA mutation^*b*^	MIC (µM)								
			DG167	INH	RIF	EMB	ETH	Moxi	BDQ	PA824	SQ109
M. tuberculosis H37Rv	None		0.39	0.19	0.001	0.78	25	0.03	1	0.1	0.8
M. tuberculosis DRM167-16x3	gTc-gCc	V123A	1.56	0.38	0.001	1.56	25	0.03	0.5	0.1	0.8
M. tuberculosis DRM167-8x6	Atg-Ttg	M213L	3.12	0.38	0.001	1.56	25	0.03	0.5	0.1	0.4
M. tuberculosis DRM167-8x3	aTc-aCc	I145T	6.25	0.38	0.002	1.56	25	0.03	1	0.1	0.8
M. tuberculosis DRM167-16x6	aTt-aGt	I122S	50	0.19	0.001	1.56	12.5	0.03	0.5	0.1	0.4
M. tuberculosis DRM167-8x2	Gcc-Acc	A119T	50	0.38	0.001	1.56	12.5	0.03	0.5	0.1	0.8
M. tuberculosis DRM167-32x11	Ggc-Agc	G240S	100	0.19	0.001	1.56	12.5	0.03	0.25	0.1	0.4
M. tuberculosis DRM167-32x2	Ccc-Acc	P206T	>100	0.19	0.001	1.56	12.5	0.03	0.12	0.1	0.4

a Each capitalized letter in the codon indicates a nucleotide change.

b Data indicate amino acid substitutions corresponding to *kasA* SNPs.

10.1128/mBio.02101-17.1 FIG S1 Docking studies performed against the previous thiolactomycin-induced conformation and our new DG167-induced conformation displayed the same trends. (A) The large docking box (the region that the ligands could explore during the AutoDock Vina calculations) is displayed as a dark purple rectangular prism. This docking box simultaneously included both substrate sites and both thiolactomycin sites in the dimer. KasA is displayed as ribbons, and the two crystallographic binding modes of DG167 per substrate site are displayed as balls and sticks. (B) The two crystallographic binding modes of DG167 are displayed as balls and sticks with dark purple carbon atoms, while the docked binding mode of DG167 is displayed as thick sticks with cyan carbon atoms. The same binding mode, with the alkyl tail buried correctly and the rings flipped to interact with the second complementary binding mode of DG167, occurred when docking against the holo structures of both PDB ID 2WGG (not displayed) and of our new DG167-induced crystal structure. (C and D) One type of crystallographic binding mode of DG167 per substrate site is treated as part of the target model and is displayed in space-filling mode with light blue carbon atoms. (C) When the “second” crystallographic binding mode of DG167 is treated as part of the target model, the “first” binding mode (with the alkyl tail buried in a deep pocket of KasA) is reproduced well by the docking calculations. The docked mode is displayed as thick sticks with green carbon atoms, while the crystallographic binding mode is rendered as balls and sticks with purple carbon atoms. (D) Similarly, when the first well-buried binding mode is treated as part of the target model, the second binding mode is reproduced by the docking calculations, albeit with some slight changes in the predicted conformation of the alkyl tail and a rotation of the sulfonyl group. The docked binding mode has dark green carbons and thick sticks, while the crystallographic binding mode is displayed as balls and sticks with magenta carbon atoms. Download FIG S1, DOCX file, 0.5 MB. Copyright © 2018 Kumar et al. 2018 Kumar et al. This content is distributed under the terms of the Creative Commons Attribution 4.0 International license.

### KasA-DG167 crystal structure reveals unique dual binding to interacting nonidentical sites in the KasA substrate-binding channel.

**FIG 1:**
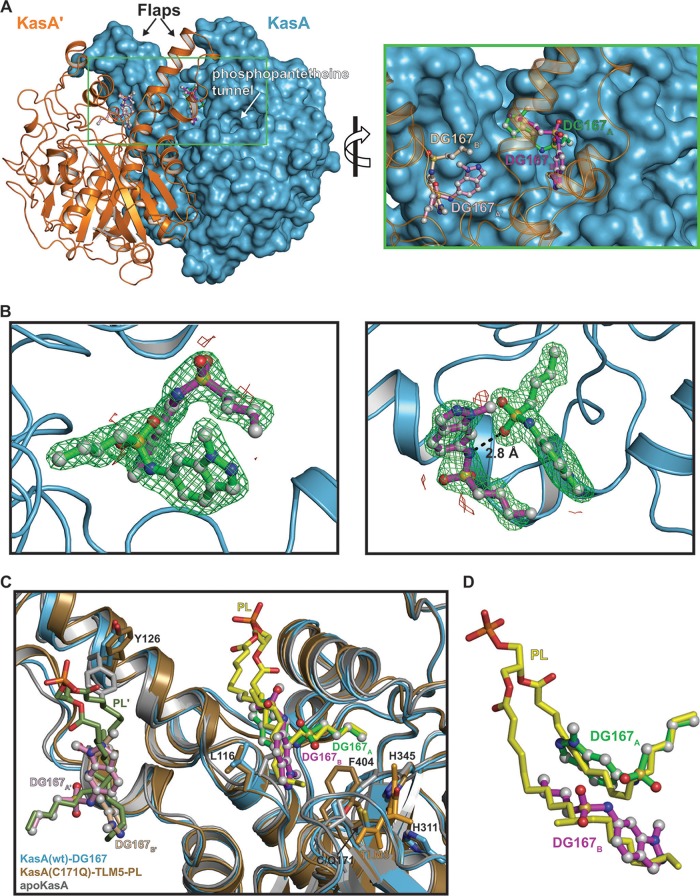
KasA-DG167 crystal structure. (A) (Left) KasA dimer with one protomer (KasA) rendered as a cyan surface and the other protomer (KasA′) rendered as an orange cartoon. The DG167 molecules are shown as ball-and-stick models with the sticks colored green (DG167_A_), magenta (DG167_B_), pink (DG167_A_′), or tan (DG167_B_′). (Right) Expanded view of area enclosed in the green rectangle in the left panel. (B) Close-up view of DG167 binding. Green mesh represents positive F_o_-F_c_ electron density scaled to 3 σ, red mesh represents negative F_o_-F_c_ electron density scaled to 3 σ, dashed lines indicate distances measured in angstroms (Å). The F_o_-F_c_ density was calculated in the absence of modeled DG167. (C) Cartoon rendering of KasA-DG167_A_/DG167_B_ (blue) aligned with apo-KasA (gray) and KasA-C171Q-TLM5-PL (tan) (PDB identifier [ID] 4C72 [23]). Phospholipid (yellow/green, PL/PL′), TLM5 (orange), and residues involved in either the catalytic triad or substrate gating are depicted as sticks and labeled black. DG167 is depicted as balls and sticks. The KasA′ protomer is hidden for clarity. wt, wild type. (D) Isolated view of DG167_A_, DG167_B_, and PL from panel C.

We solved the X-ray crystal structure of KasA in complex with DG167 to gain insights into the DG167 mechanism of action. M. tuberculosis KasA was overexpressed and purified from M. smegmatis, and the KasA-DG167 cocrystal structure was refined to 2.00-Å resolution (Fig. 1A to C; see also Table S1 in the supplemental material). We also determined the crystal structure of apo-KasA, which was refined to 1.80-Å resolution using crystallization conditions similar to those used to crystallize KasA-DG167 (Fig. 1C; see also Table S1). We found that each KasA monomer bound two DG167 molecules (KasA-DG167_2_), with the result that the biological KasA dimer bound four DG167 molecules, i.e., KasA binds DG167_A_ and DG167_B_ and KasA′ binds DG167_A_′ and DG167_B_′ (Fig. 1A to C). Note that our KasA-DG167_2_ structure is not congruent with the structure in the study reported by Abrahams et al., where one molecule of DG167 and one molecule of polyethylene glycol (PEG) were modeled and refined in each KasA subunit (25). While their different density map suggested the presence of a “PEG-like molecule,” their crystallization conditions excluded PEG. This, together with our data, suggested that the PEG was misassigned. More specifically, in our KasA-DG167_2_ structure, in place of what was modeled as PEG by Abrahams et al., a second molecule of DG167 is present. Thus, as detailed below, our KasA-DG167_2_ structure uncovers a previously unknown DG167-binding site and reveals an intermolecular interaction between the two DG167 molecules bound to nonidentical sites.

10.1128/mBio.02101-17.8 TABLE S1 Data collection and refinement statistics. Download Table S1, DOCX file, 0.02 MB. Copyright © 2018 Kumar et al. 2018 Kumar et al. This content is distributed under the terms of the Creative Commons Attribution 4.0 International license.

**FIG 2:**
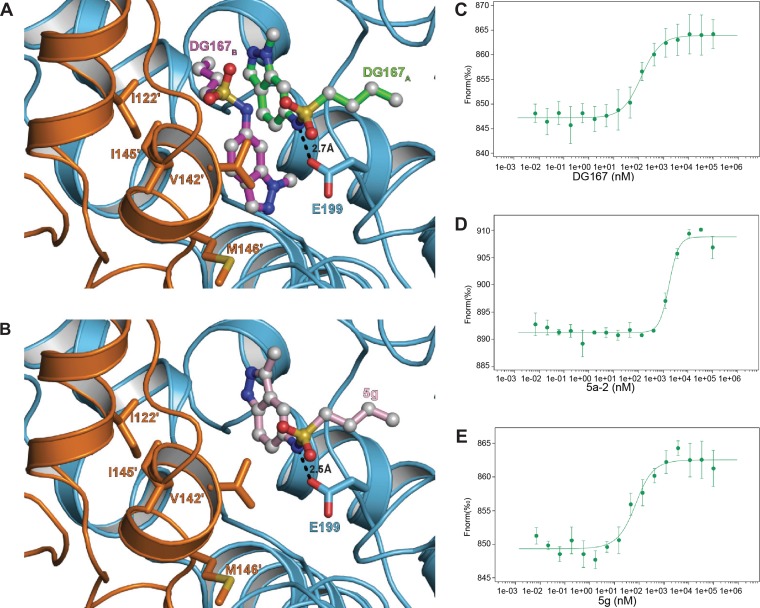
Analysis of DG167 analogues binding to KasA. (A and B) Cartoon representation of two DG167 molecules (DG167_A_ [green sticks] and DG167_B_ [magenta sticks]) or one molecule of 5g (pink sticks) binding to a KasA dimer with one protomer colored cyan (KasA) and the other colored orange (KasA′). In panel A, the KasA′ residues that interact with DG167 are depicted as orange sticks. These residues are similarly displayed in panel B but make no contacts to the analog 5g. Dashed lines indicate bond distances measured in angstroms. (C to E) MST quantification for DG167, its transposed indazole analogue 5g, and its inactive des-methyl analog 5a-2 binding to KasA. DG167, 5a-2, and 5g were titrated at between 0.007 nM and 100,000 nM with 49.5 nM labeled KasA. DG167 binds to KasA with an EC_50_ of 130.9 ± 18.2 nM. 5a-2 binds to KasA with an EC_50_ of 1,736.1 ± 221.2 nM. 5g binds to KasA with a *K_d_* = 46.5 ± 18.7 nM.

Examination of the clearly interpretable electron density data corresponding to DG167_A_ and DG167_B_ showed that these two DG167 molecules bound to nonoverlapping sites in the KasA acyl channel identified as the phospholipid (PL)-binding site observed in previous crystal structures (28) (Fig. 1A to C; see also Fig. S2A to C) (29). Consistent with the KasA-DG167_2_ structure, the KasA mutations identified in the laboratory-generated DG167-resistant M. tuberculosis strains mapped near the binding sites of both DG167_A_ and DG167_B_ (Fig. S3). Interestingly, we found that the aliphatic moiety of DG167_A_ mimics binding of the PL acyl tail as it inserts into a pocket formed by residues Gly200, Ile202, Pro206, Phe239, His345, and Ile347 (Fig. 1C and D; see also Fig. S2A to C). The DG167_B_ aliphatic moiety similarly follows the path traced by PL and presumably bona fide acyl substrates (28). The DG167_A_ and DG167_B_ indazole groups make hydrophobic interactions throughout the acyl channel, and the DG167_B_ indazole group mediates additional hydrophobic contacts across the KasA/KasA′ dimer interface. The KasA-DG167_2_ interaction is further stabilized by hydrogen bonds between the DG167_A_ sulfonamide N-H and Glu199 (Fig. S2A) as well as by the DG167_B_ indazole group nitrogen and a water molecule coordinated by residues Gly115, Asn148, and Ala170 (Fig. S2A and B). Importantly, the two molecules of DG167 bound to the KasA monomer form an intermolecular hydrogen bond. Specifically, a DG167_A_ sulfonamide oxygen hydrogen bonds with the DG167_B_ sulfonamide N-H (Fig. 1B and Fig. 2A; see also Fig. S2B). In sum, an extensive array of both hydrophobic interactions and hydrogen bonds stabilize the binding of two DG167 molecules to a KasA monomer. Moreover, the two DG167 molecules hydrogen bond to each other and occupy the KasA surface that would otherwise bind the elongating acyl chain prior to the condensation reaction catalyzed by KasA. Thus, the KasA-DG167_2_ cocrystal structure represents a plausible mechanism by which DG167 can effectively compete for substrate binding, inhibit KasA activity, and produce a bactericidal effect on M. tuberculosis.

10.1128/mBio.02101-17.2 FIG S2 Schematic representation of (A) DG167_A_-DG167_B_, and DG167_A_-KasA interactions. (B) DG167_A_-DG167_B_, DG167_B_-KasA, and DG167_B_-KasA′ interactions; and (C) KasA-PL interactions in PDB ID 4C72 chain A (23). Molecules are labeled consistently throughout the figure—DG167_A_ is depicted as green bonds, DG167_B_ is depicted as magenta bonds, phospholipid (PL) is depicted as yellow bonds, and hydrogen bonds are depicted as dashed lines measured in angstroms (Å). (A) The blue semicircles with radiating lines represent hydrophobic contacts mediated by KasA residues and DG167_A_. (B) The blue semicircles with radiating lines represent hydrophobic contacts mediated by KasA residues and DG167_B_, while orange semicircles with radiating lines represent hydrophobic contacts mediated by KasA′ residues and DG167_B_. (C) The blue semicircles with radiating lines represent hydrophobic contacts mediated by KasA residues and PL, while orange semicircles with radiating lines represent hydrophobic contacts mediated by KasA′ residues and PL. PL-binding residues surrounded with a green, magenta, or red line represent residues from the KasA-DG167 structure that interact with DG167_A_ or DG167_B_ or both, respectively. The schematic was produced with LIGPLOT (29). Download FIG S2, DOCX file, 5.9 MB. Copyright © 2018 Kumar et al. 2018 Kumar et al. This content is distributed under the terms of the Creative Commons Attribution 4.0 International license.

10.1128/mBio.02101-17.3 FIG S3 Proximity of DG167 to the KasA DG167-resistant mutations. A KasA biological dimer with one protomer is rendered as a cyan cartoon (KasA), and the other protomer is rendered as an orange surface (KasA′). The positions of mutations conferring resistance to DG167 (Table 2) are highlighted as red sticks on KasA and as a yellow surface on KasA′. The DG167 molecules are depicted as ball-and-stick models with the bonds colored green (DG167_A_), magenta (DG167_B_), pink (DG167_A_′), or tan (DG167_B_′). Download FIG S3, DOCX file, 4.3 MB. Copyright © 2018 Kumar et al. 2018 Kumar et al. This content is distributed under the terms of the Creative Commons Attribution 4.0 International license.

### X-ray crystallographic analysis shows that dual inhibitor binding is not required to disrupt KasA function.

While two molecules of DG167 occupy the acyl channel, we tested the hypothesis that a single compound bound to the channel would be sufficient to block chain elongation. Structure-based design was used to arrive at DG167 analog 5g that was optimized to bind at the KasA DG167_A_ binding site. 5g showed 2× improved MIC compared to DG167 (0.2 µM; Table 3). We determined the X-ray crystal structure of KasA in complex with 5g (Fig. 2A and B) (Table 3; see also Table S2), which revealed that 5g bound the acyl channel only once at the DG167_A_ binding site. We postulate that 5g cannot bind in place of DG167_B_ because a similar pose at this second binding site would force the 5g indazole N (1)-H to interact with a hydrophobic surface across the KasA dimer interface.

**TABLE 3:**
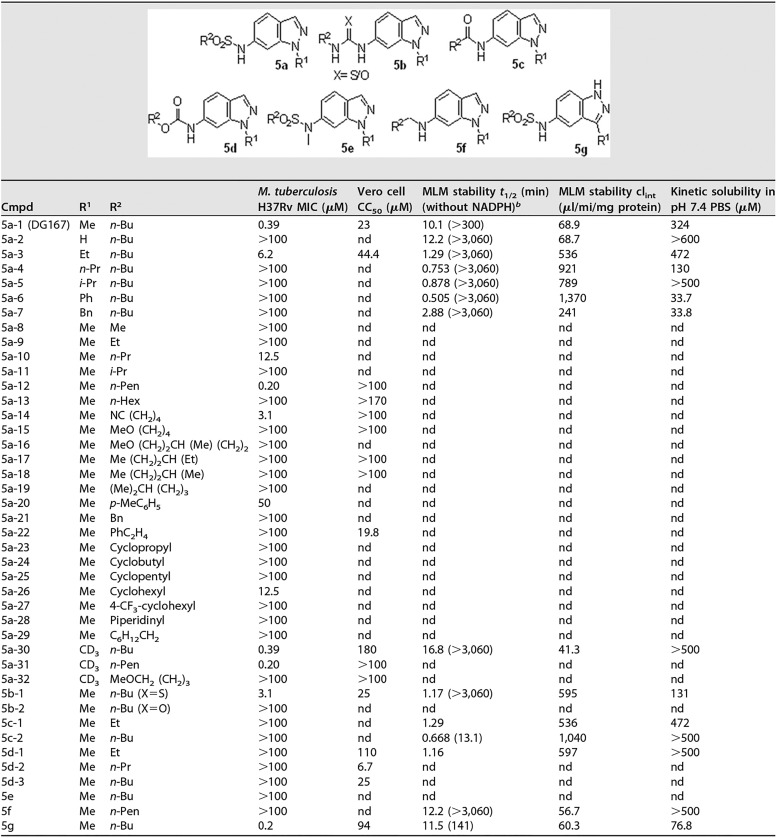
Structure-activity relationships for DG167 analogs^*a*^

a cl_int_, intrinsic clearance; nd, not done; Me, methyl; Et, ethyl; Bu, butyl; Pr, propyl; Pen, pentyl; Hex, hexyl; Bn, benzyl; MeO, methoxy.

b Data corresponding to MLM *t*_1/2_ (in minutes) without NADPH are shown in parentheses.

10.1128/mBio.02101-17.9 TABLE S2 (A) List of genes showing a ≥2.0-fold transcription change with *P* value (<0.05) upon treatment with INH or DG167 or INH plus DG167. (B) List of 32 genes upregulated (>2 fold) with combination treatment with INH plus DG167. (C) List of 22 genes downregulated (>2 fold) with combination treatment with INH plus DG167. Download Table S2, XLSX file, 0.05 MB. Copyright © 2018 Kumar et al. 2018 Kumar et al. This content is distributed under the terms of the Creative Commons Attribution 4.0 International license.

### Biochemical solution studies to quantify binding affinity of inhibitors with KasA.

Other studies have described a complex coupled assay that uses artificial substrates and multiple enzymes to study KasA inhibition *in vitro* (30). Here, we developed a microscale thermophoresis (MST) assay to measure the binding affinity of DG167 and its analogs for KasA and determined that the KasA-DG167 interaction has a 50% effective concentration (EC_50_) of 130.9 ± 18.2 nM (Fig. 2C). In comparison, 5g bound KasA tightly, with a dissociation constant (*K_d_*) of 46.5 ± 18.7 nM (Fig. 2D), while an inactive des-methyl analog, 5a-2, bound KasA comparatively weakly, with an EC_50_ of 1,736.1 ± 221.2 nM (Fig. 2E).

### DG167 inhibits mycolic acid biosynthesis.

**FIG 3:**
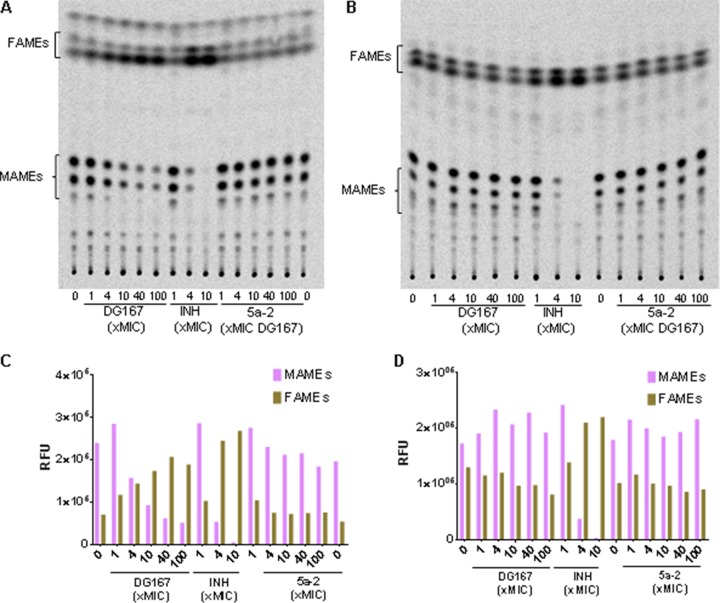
DG167 inhibits mycolic acid biosynthesis *in vivo.* (C and D) Normal-phase TLC analysis of MAMEs and FAMEs from wild-type M. tuberculosis (H37Rv) and DG167-resistant M. tuberculosis (isolate DRM167-32x2; Table 1). The cultures were treated with increasing concentrations of DG167 and inactive analog 5a-2. Total lipids were extracted, subjected to methyl esterification after [^14^C]acetate incorporation, and resolved by TLCs. RFU, relative fluorescence units. (A) M. tuberculosis H37Rv. (B) M. tuberculosis DRM167-32x2 isolate. (C) Densitometric analysis of MAMEs and FAMEs from panel A using ImageQuant (GE Healthcare). (D) Densitometric analysis of MAMEs and FAMEs from panel B. Equal counts (20,000 cpm) were loaded, and the TLC was developed using hexane/ethyl acetate (19:1 [vol/vol], 2 runs).

KasA is an essential component of the FAS-II pathway along with InhA. We studied the effect of DG167 on mycolic acid biosynthesis using a radiolabeled precursor, [^14^C]acetate. DG167 exhibited dose-dependent inhibition of mycolic acid biosynthesis (Fig. 3A and C) like that seen with INH, and inactive des-methyl analog 5a-2 (Table 3) did not show any inhibition. Furthermore, a DG167-resistant isolate harboring KasA_P206T_ (DRM167-32x2; Table 2) was found to be resistant to DG167-mediated inhibition of mycolic acid biosynthesis (Fig. 3B and D). Additionally, inhibitors of FAS-II like INH are known to cause accumulation of FAS-I products; also, DG167 treatment caused accumulation of C_16_-C_26_ short-chain fatty acids (data not shown), confirming specific inhibition of FAS-II by DG167.

### Efficacy of DG167 and INH in combination.

**FIG 4:**
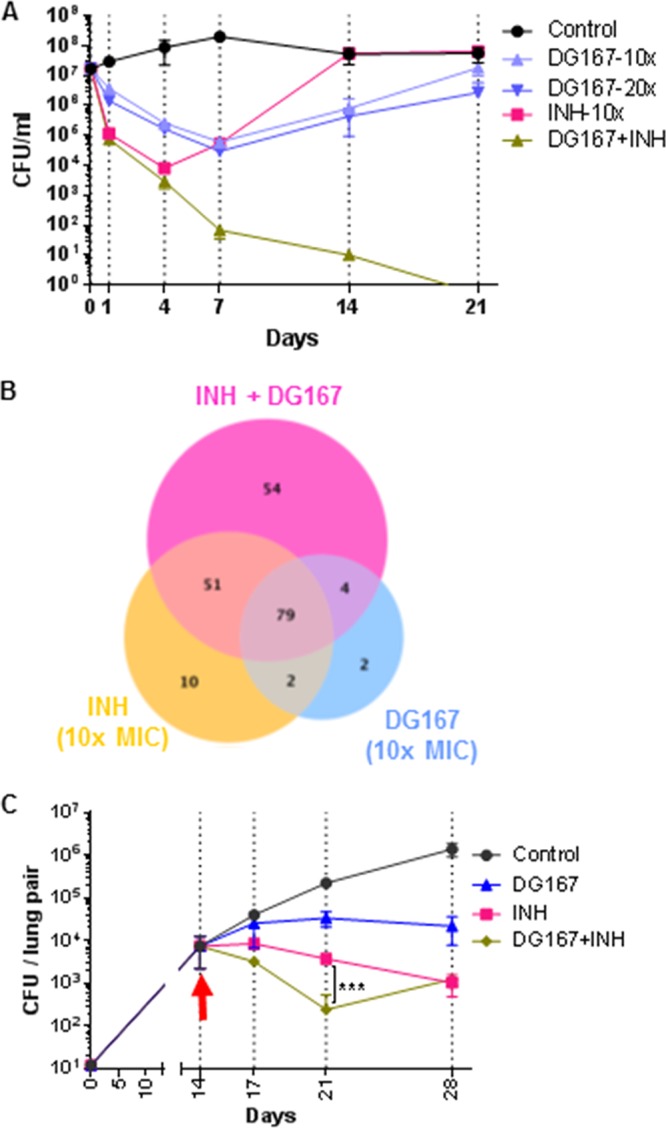
DG167 synergizes with INH *in vitro* and *in vivo.* (A) *In vitro* killing curves for M. tuberculosis strain H37Rv after incubation with given concentrations of DG167 or INH or a combination of those drugs. Killing activity was monitored by plating for CFU. (B) Venn diagram showing differential gene expression data from an RNA-seq experiment upon treatment with 10× DG167 or 10× INH or the drug combination. (C) *In vivo* efficacy of DG167 and INH alone and in combination in an acute model of M. tuberculosis infection in mice. The arrow indicates the day when treatment was started. The significance was determined using the Kruskal-Wallis test. On days 21 and 28, all treatments showed significant effects compared to the control (*P* = <0.0001).

INH treatment is known to induce high-level *kasA* expression in M. tuberculosis (31, 32), which could potentially antagonize the activity of DG167. However, a checkerboard assay revealed that the effects of DG167 and INH were not antagonistic but additive (summation fractional inhibitory concentration [ΣFIC] = 1). We next evaluated the bactericidal activity of the combination of DG167 and INH. M. tuberculosis cultures (∼10^7^ cells/ml) were treated with 10× and 20× the MIC of DG167 or with 10× the MIC of INH or with a combination of the two drugs at 10× the MIC of each compound (Fig. 4A). Treatment with DG167 alone reduced viable CFU levels by 2 log_10_ over 7 days, and this bactericidal effect was independent of the DG167 concentration tested. Treatment of the cultures with INH alone (10× MIC) resulted in rapid reduction of viable CFU levels followed by rapid regrowth; higher INH concentrations are also known to produce similar kill curves. This typical pattern of killing and regrowth has been previously attributed to the emergence of persisters combined with the emergence of INH-resistant clones (11, 33). Interestingly, the use of DG167 and INH in combination markedly improved upon the bactericidal activity of either drug used alone, as the combination produced a rapid reduction in CFU, leading to complete culture sterilization. We confirmed that the synergistic lethality (34) that we observed with the treatment using DG167 and INH in combination was specific to KasA inhibition by repeating the time-kill studies using a DG167-resistant isolate (M. tuberculosis DRM-32x2) (Table 2). In this setting, the time-kill kinetics of INH plus DG167 were comparable to those seen with cultures treated with INH alone (Fig. S4A). This strongly suggests that INH/DG167 synergistic lethality requires KasA inhibition. Finally, to assess the effect of combination treatment with INH and DG167 on persister populations, we tested INH and DG167 alone or in combination using the Hu-Coates 100-day-old-culture model (35, 36). Interestingly, combined treatment with INH and DG167 (using a 100 µM concentration of each drug) produced a >2 log_10_ reduction in these persister cultures. In contrast, persister cultures treated with a 200 µM concentration of either INH or DG167 alone showed minimal killing (Fig. S4B). This suggests that the synergistic lethality that we observed is likely attributable to increased activity against persister cells.

10.1128/mBio.02101-17.4 FIG S4 (A) Susceptibility of DG167-resistant strain to dual treatment. DRM167-32x2 was treated with INH or DG167 or INH plus DG167 at 10× the MIC of each drug or with DMSO (control). Growth was measured by determination of the OD_595_ at given time points. (B) Hu/Coates 100-day-old-culture model of persistence. The 100-day-old cultures were treated with INH (200 µM), DG167 (200 µM), INH plus DG167 (100 µM [each]), or PA824 (100 µM). (C and D) Macrophage intracellular inhibition assay. J774A.1 cells were infected with M. tuberculosis (mc26206) expressing luciferase at a multiplicity of infection (MOI) of 1. Levels of inhibition of intracellular survival and growth were determined by measuring (C) luciferase activity with a luminometer and (D) CFU 48 h after drug was added. Download FIG S4, DOCX file, 0.05 MB. Copyright © 2018 Kumar et al. 2018 Kumar et al. This content is distributed under the terms of the Creative Commons Attribution 4.0 International license.

### Transcriptional profiling of M. tuberculosis treated with DG167 and INH reveals a unique signature that correlates with *in vitro* synergistic lethality.

We performed transcriptome sequencing (RNA-seq) analysis to further understand the molecular mechanism of action of DG167 and its interaction with INH. Single-drug treatment performed with either INH or DG167 strongly induced expression of the *kasA*, *kasB*, and *acpM* genes and the *iniBAC* operon and significantly altered the transcription of other genes known to be modulated by INH (26, 37, 38) (Table S2). These findings demonstrate the potent activity of DG167, which has a submicromolar MIC despite the fact that it strongly induces its own cellular target (KasA). In contrast, clinical resistance to INH can be caused by mutations in the *mabA-inhA* promoter that increase *inhA* expression only moderately (32). With respect to the dual treatment with DG167 and INH, all but six of the genes that were differentially expressed in response to the DG167 treatment were also differentially expressed in response to the INH treatment, further supporting the hypothesis that DG167 and INH inhibit targets essential for the same biosynthetic pathway (Fig. 4B). As expected, dual treatment with DG167 and INH altered the expression of almost all (*n* = 79) of the genes that were differentially expressed by treatment with each drug alone. However, a surprisingly large number of 54 additional “dual-drug-modulated” genes were differentially expressed (32 genes were upregulated and 22 were downregulated) only in the dual-treated cultures (Fig. 4B). The putative or known functions of this gene set are shown in Table S2B and C. (39). Lists of genes showing differential regulation with single-drug treatments are provided for comparison (Table S2D and E). The differential expression of these 54 dual-drug-modulated genes correlated with synergistic lethality, i.e., a loss of persisters and culture sterilization, rather than with drug exposure. A total of 32 of the unique dual-drug-modulated genes were upregulated. This set of upregulated genes was enriched in those encoding oxidoreductases and putative transposase elements (Table S2B). Twenty-two of the unique dual-drug-modulated genes were downregulated (Table S2C). This set of downregulated genes was enriched for chaperones (*groEL1*, *groEL2*, and *groES*), which are typically upregulated after treatment with bacteriostatic drugs (40–42) and are also upregulated in M. tuberculosis persisters during INH exposure. Together, these results suggest that combined treatment with both DG167 and INH activates a cellular response associated with loss of persistence and induction of cidality that is distinct from the cellular response induced by single-drug treatment.

### DG167 profiling.

We profiled DG167 for desirable drug-like and pharmacokinetic (PK) properties. DG167 had a good selectivity index (SI; 50% cytotoxic concentration [CC_50_]/MIC) of 59 with Vero cells (Table S3). The kinetic solubility in phosphate-buffered saline (PBS) (pH 7.4) was 324 μM. The Caco-2 permeabilities (P_A-B_ and P_B-A_) were 71.8 × 10^−6^ and 45.6 × 10^−6 ^cm/s, respectively. Cytochrome P450 (CYP) inhibition studies (Table S3) demonstrated DG167 did not significantly inhibit CYP enzymes except CYP2C19 (50% inhibitor concentration [IC_50_] = 12 μM), and hERG inhibition (IC_50_ > 20 μM) was also ruled out. Mouse liver microsomal (MLM) stability was suboptimal, with a half-life (*t*_1/2_) of 10.1 min. However, the MLM *t*_1/2_ in the absence of NADPH (to exclude oxidative metabolism) was >300 min. We examined MLM-generated metabolites through mass spectrometry (MS) to identify metabolic liabilities. A demethylated species, corresponding to loss of the 1-methyl group (Fig. S5A), predominated among the metabolites. When synthesized (5a-2) (Table 3), this metabolite lacked activity against M. tuberculosis (MIC > 100 µM), suggesting a metabolic liability at a position that is necessary for whole-cell activity. Since DG167 was easily demethylated into an inactive form in MLM, we embarked upon experiments designed to reveal its metabolism within M. tuberculosis. Promisingly, DG167 accumulated in a dose-dependent manner in M. tuberculosis cells (Fig. S5B), and the demethylated form was not detected, indicating that DG167 is not inactivated by demethylation inside the bacteria. The inactive des-methyl analog (5a-2) also showed dose-dependent accumulation inside M. tuberculosis, confirming that intact DG167 was essential for target inhibition and whole-cell activity (Fig. 4B) (43).

10.1128/mBio.02101-17.5 FIG S5 DG167 metabolism and uptake. (A) DG167 metabolites in mouse liver microsomes. Demethylation (peak M5) of DG167 was determined to be major product of MLM metabolism. (B) DG167 uptake in M. tuberculosis. The M. tuberculosis cultures grown on filters were treated with increasing concentrations of DG167 and its demethylated analog (5a-2) on solid agar plates. After 24 h of treatment, the metabolites were extracted and analyzed by LC-MS as described in Materials and Methods. Download FIG S5, DOCX file, 0.2 MB. Copyright © 2018 Kumar et al. 2018 Kumar et al. This content is distributed under the terms of the Creative Commons Attribution 4.0 International license.

10.1128/mBio.02101-17.10 TABLE S3 Profile of DG167. Download Table S3, DOCX file, 0.02 MB. Copyright © 2018 Kumar et al. 2018 Kumar et al. This content is distributed under the terms of the Creative Commons Attribution 4.0 International license.

### Synthesis of DG167 and analogs.

The synthesis of DG167 and a focused series of analogs are depicted in the supplemental material (Fig. S6). To address the issue of the primary metabolic stability through demethylation, a series of N1-substituted indazoles was synthesized and evaluated for antitubercular activity and MLM stability. In comparison to DG167, longer or branched alkyl chains, with the exception of an ethyl group at the N1 position, had unfavorable effects on both activity and MLM *t*_1/2_. The trideuteriomethyl analog 5a-30 offered an improvement in metabolic stability (*t*_1/2_ = 16.8 min). Since 5a-30 was a close analog of DG167, we also determined its cocrystal structure with KasA and found that it also exhibited a binary binding mode consistent with DG167 (Fig. S7). Analogs featuring replacement of the 6-position sulfonamide with functional groups such as the carbamate, amide, amine, and urea/thiourea groups, while retaining the 1-N-methyl group, were synthesized and assayed. Among them, only 1-*n*-butyl-3-(1-methyl-1*H*-indazol-6-yl)thiourea (5b-1) demonstrated modest activity (MIC = 3.1 μM). *N*-methylation of the sulfonamide NH of DG167 (5e) resulted in loss of activity. Based on the results described above, N1-methyl substitution and a 6-sulfonamide were identified as critical elements for whole-cell efficacy. Subsequently, the sulfonamide *n*-butyl substituent was truncated or cyclized. Again, a loss of whole-cell activity was noted. Furthermore, the significant loss of whole-cell efficacy for analogs with branched alkyl sulfonamide substituents (i.e., 5a-16 to 5a-19) hinted at the specific steric requirements of the target enzyme binding site. While the *n-*hexyl sulfonamide analog 5a-13 was inactive, the elongation of *n*-butyl to an *n*-pentyl chain at the R^2^ position increased the activity over the level seen with the parent by 2-fold when R^1^ = methyl (5a-12) as well as *d_3_* = methyl (5a-31). Finally, the indazole’s pyrazole unit was transposed to afford 5g, which demonstrated a 2× improvement in whole-cell activity. A DG167-resistant isolate (M. tuberculosis DRM-32x2; Table 2) was tested against active analogs of DG167, and we observed resistance (MIC > 100 μM), confirming the target specificity of the analogs.

10.1128/mBio.02101-17.6 FIG S6 Synthetic scheme for DG167 and its analogs. The *N*-methylation of commercially available 6-nitro-1*H*-indazole afforded a mixture of 1-methyl-6-nitro-1*H*-indazole and 2-methyl-6-nitro-1*H*-indazole. The chromatographically separable 1-methyl-6-nitro-1*H*-indazole was reduced, and then the free amine was derivatized using one of four different methods (depending on the desired functional group) to generate the analytically pure compounds. The parent compound (DG167) was synthesized by treating 6-amino-1-methyl-1*H*-indazole with butane-1-sulfonyl chloride in the presence of pyridine. Reagents and conditions were as follows: (a) (i) NaH, dimethylformamide (DMF), 0°C, 30 min; (ii) R^1^I, DMF, room temperature (RT), 16 h or R^1^I (1.2 eq), CuI (0.05 eq), K_3_PO_4_ (2 eq), *N*,*N*-dimethylethylenediamine (0.1 eq), DMF, 110°C, 72 h; (b) 10 wild-type (wt) percent Pd/C, HCOONH_4_, ethanol (EtOH), RT, 4 h; (c) R^2^SO_2_Cl, pyradine (py), RT, 16 h; (d) (i) NaH, DMF, 0°C, 30 min, (ii) CH_3_I, DMF, RT, 16 h; (e) R^2^NCS/R^2^CNO, triethylamine (TEA), dichloromethane (DCM), 40°C, 16 h; (f) (i) 1,1' carbonyldiimidazole, DCM, 35°C, 4 h; (ii) R^2^OH, DCM, 35°C, 16 h; (g) R^2^COCl, py, RT, 16 h; (h) LiAlH_4_, tetrahydrofuran (THF), 4 h. Download FIG S6, DOCX file, 2.0 MB. Copyright © 2018 Kumar et al. 2018 Kumar et al. This content is distributed under the terms of the Creative Commons Attribution 4.0 International license.

10.1128/mBio.02101-17.7 FIG S7 KasA-5a-30 crystal structure. (A) (Left) KasA dimer with one protomer (KasA) rendered as a green surface and the other protomer (KasA′) rendered as a red cartoon. The 5a-30 molecules are shown as ball-and-stick models, with the sticks colored purple (5a-30_A_), red (5a-30_B_), cyan (5a-30_A_′), or magenta (5a-30_B_′). (Right) Expanded view of the area enclosed in the black rectangle in the left panel. (B) Cartoon rendering of KasA-5a-30_A_/5a-30_B_ (green) aligned with apo-KasA (grey) and KasA-C171Q-TLM5-PL (tan); PDB ID 4C72 (23). Phospholipid (yellow/olive, PL/PL′), TLM5 (orange), and residues involved in either the catalytic triad or substrate gating are depicted as sticks and labeled black. 5a-30 is depicted as balls and sticks. The KasA′ protomer is hidden for clarity. Download FIG S7, DOCX file, 2.0 MB. Copyright © 2018 Kumar et al. 2018 Kumar et al. This content is distributed under the terms of the Creative Commons Attribution 4.0 International license.

### Mouse pharmacokinetic profile and dose tolerability.

We performed PK profiling to facilitate *in vivo* efficacy studies. The PK profile of DG167 administered as a single oral dose of 25 mg/kg of body weight revealed promising oral bioavailability (92.3%) and plasma levels (area under the concentration-time curve from 0 to time *t* [AUC_0–_*_t_*] of 8,083.96 h*ng/ml; Table S3). Plasma levels were maintained above the MIC for over 4 h (Table S3 and data not shown). At a intravenous (i.v.) dosage of 5 mg/kg, the half-life was 0.33 h. A dose escalation study was performed at 50, 100, 250, and 500 mg/kg where the mice were monitored for 8 h. Doses of both 50 and 100 mg/kg were well tolerated. At the higher doses, the mice exhibited heavy breathing, hunched posture, and decreased activity. Mice subjected to dose tolerability studies performed for 5 days at 50 mg/kg and 100 mg/kg and a combination of DG167 (100 mg/kg) with INH (25 mg/kg) did not show any behavioral changes or weight loss, and normal liver pathology was observed.

### DG167 and INH exhibited synergy in *in vitro* intracellular and *in vivo* infection models.

In M. tuberculosis-infected J774A.1 macrophage cells, DG167 showed synergistic killing of M. tuberculosis with INH compared to either INH or DG167 alone (Fig. S4C and D). We then studied the activity of both compounds using an acute murine infection model. INH is highly active against M. tuberculosis in the acute model, typically showing rapid clearance with 2 weeks of treatment. Therefore, we also studied treatment efficacy at the earlier time points (day 3 and day 7 posttreatment). At those two time points, INH plus DG167 showed a much greater reduction in bacterial burden than either compound alone (Fig. 4C). By 2 weeks of treatment, the combined regimen was no better than INH alone; however, the number of CFU detected for either of these treatment conditions was close to the lower limit of detection by this time point, making an improvement resulting from the combined treatments difficult to detect. These data further emphasize the potential benefits for treating TB with INH combined with a KasA inhibitor.

## DISCUSSION

Our study demonstrated that DG167 inhibits mycolic acid biosynthesis by targeting KasA, an essential member of the FAS-II complex in M. tuberculosis. DG167 possessed *in vitro* activity comparable to that of INH, and it targeted the same cyclic pathway producing long-chain mycolic acids. INH and DG167 showed similar three-phase kill curves *in vitro*, characterized by an initial killing phase followed by a plateau in CFU and, finally, outgrowth of both drug-susceptible and drug-resistant bacteria. Combined treatment with INH and DG167 eliminated the undesirable second and third phases and substantially enhanced bactericidal activity, leading to complete sterilization of the bacterial cultures. This observed lethality may extend to eradication of persister M. tuberculosis cells, as demonstrated by the synergy exhibited between INH and DG167 in the Hu-Coates 100-day-old-culture model. KasA is a proven essential gene and a putative drug target in M. tuberculosis; however, its expression is greatly induced by FAS-II inhibitors such as INH and ETH suggesting a network of molecular interactions that may affect the efficacy of drug combinations, a key aspect of multidrug TB therapy. Thus, it was an open issue as to whether KasA inhibition would be synergistic or antagonistic with INH. Our results confirm the former activity by demonstrating synergistic lethality between INH and DG167. The term “synergistic lethality” has been used to describe situations where two bacteriostatic drugs exhibit bactericidal properties when used together (34). We believe that this term is also appropriate to describe the combined effect of DG167 and INH on M. tuberculosis (39).

Our study data suggest that dual treatment with DG167 and INH is likely to activate intrabacterial processes that are associated with enhanced cidality. The pattern of genes differentially expressed by dual INH/DG167 treatment compared to treatment with either compound administered individually, including the induction of oxidoreductases and nitrate reductase, and the suppression of molecular chaperones suggest that dual treatment activates bactericidal and represses persistence mechanisms within the cell. Our premise is supported by studies in Escherichia coli that have shown that bactericidal drugs develop common metabolic signatures after the first 30 min of treatment such as elevation of central carbon metabolites, breakdown of the nucleotide pool, and elevated redox state, i.e., increased respiration. This signature differs from those common to bacteriostatic drugs, e.g., accumulation of metabolites that feed the electron transport chain and suppress respiration (40, 42).

As described here and by others (25), DG167 has a number of favorable pharmacokinetic/pharmacodynamic (PK/PD) properties, including high potency, solubility, and selectivity and low protein binding, suggesting that this molecule is a promising drug discovery lead. However, DG167 has relatively poor MLM stability, an attribute that requires improvement. We expect that the 2.0-Å KasA-DG167 (and the 1.8-Å KasA-5a-30) structures described here will guide medicinal chemistry efforts. Crystal structures have already revealed that DG167 differs from other previously described KasA inhibitors such as thiolactomycin (TLM) and its analogs (e.g., TLM5) that function by binding to the KasA catalytic site (Fig. 1C). Instead, DG167 demonstrates what we believe is a unique property for an antibacterial in that two DG167 molecules bind to nonidentical and nonoverlapping surfaces of their target. Furthermore, DG167_A_ and DG167_B_ form an intermolecular hydrogen bond. It is also important that the KasA biological unit is a homodimer, and, as observed in the crystal structure via the application of crystallographic symmetry, there are four molecules of DG167 bound per biological KasA homodimer. DG167 binding stabilizes the KasA acyl channel flaps that are otherwise disordered in the absence of PL or DG167 (Fig. 1A). In addition to interacting with the respective KasA, DG167_B_′ interacts with the KasA acyl channel flap, and DG167_B_ interacts with the KasA′ acyl channel flap. Similarly, the KasA acyl channel flaps are ordered in the KasA-5g crystal structure, and 5g does not make contacts across the dimer interface. Thus, it seems that the requirement for KasA acyl channel flap stabilization is acyl channel occupancy, and it seems likely that flap stabilization contributes allosterically to the binding of DG167 or 5g to KasA. In addition, while we observed two bound DG167 molecules in the KasA-DG167_2_ structure, we hypothesize that the binding of either DG167 molecule would be sufficient to block acyl chain elongation. This hypothesis is supported by the fact that the DG167 analog 5g is an active inhibitor that binds only once to the acyl channel in a conformation similar to that seen with DG167_A_. Similarly, we speculate that combinations of different DG167 analogs, e.g., one that binds tightly to the DG167_A_ site and another that binds tightly to the DG167_B_ site, may together have improved whole-cell activity and KasA inhibition characteristics relative to a single-DG167 analog.

Our structural studies revealed other unique features of DG167 that might prove useful in the design of KasA inhibitors. In current models, acyl-AcpM drives a conformational change in Phe404 that not only activates catalysis by triggering proton transfer from Cys171 to His311 but also permits acyl chain access to the acyl channel and causes the rearrangement of additional gatekeeper residues. In the KasA-DG167_2_ structure, Phe404 is in the closed conformation; i.e., the acyl chain is prevented from entering the channel. To our knowledge, this is the first time that KasA has been shown to bind a ligand (e.g., inhibitor or PL) while Phe404 is in the closed conformation. Moreover, it is clear that DG167 and the acyl chain could not bind simultaneously to KasA. Therefore, DG167 would bind preferentially to nonacylated KasA, which distinguishes DG167 from previously identified KasA inhibitors that bind preferentially to acylated KasA. This is, in part, what makes DG167 unique and different from cellular free fatty acids, which could, in theory, disrupt KasA function similarly to DG167 if binding were promiscuous. While KasA has evolved mechanisms to prevent entry of free fatty acids into the acyl channel via the phosphopantetheine tunnel and the surface near the disordered flaps, DG167 circumvents the requirement for both AcpM and the opening of gatekeeper residue Phe404.

In a previously reported structure of KasA-DG167, PEG was modeled in the electron density at a position equivalent to that of DG167_B_ (25) (Fig. 1B). As PEG was not included during their experiment, those authors speculated that the modeled PEG may represent a contaminant from adjacent wells. They alternatively propose that the molecule may represent a residual lipid rather than PEG but acknowledge that even apo-KasA structures crystallized using PEG as a precipitant adopt the closed conformation and do not contain lipid at this position. We concur that modeling DG167_B_ with respect to their electron density was not justified. Possible explanations accounting for the differences between our KasA-DG167_2_ structure and their KasA-DG167 structure include their lower resolution diffraction, twinned crystals, different crystallographic space group, and cocrystallization procedures. We conclude that the KasA acyl tunnel can bind two molecules of DG167 and that, while either molecule may be sufficient to disrupt KasA function, the DG167-DG167 binding energy may make important additional contributions to KasA inhibition.

The conformational constraints and molecular interactions that govern the interactions between DG167 and KasA also suggest modifications that could either improve the potency or permit modifications that increase metabolic stability of the current DG167 lead. In fact, the observed SAR trend for our initially synthesized analogs can be explained based on the KasA-DG167 crystal structure. Consistent with the two DG167 molecules interacting via a hydrogen bond formed between the sulfonamide oxygen on DG167_A_ and the sulfonamide NH of DG167_B_, methylation of this nitrogen (5e) resulted in a loss of activity. These substitutions for the sulfonamide would also seem likely to significantly alter placement of the pendant alkyl chain. Carbamate, amide, amine, and urea/thiourea functionalities at position 6 of the indazole also disrupt this intermolecular H-bonding interaction and lead to abrogation of activity. Truncation of the sulfonamide alkyl chain (5a-8 to 5a-10) or adding bulky/branched substituents (5a-11 and 5a-16 to 5a-29) may disrupt placement of the acyl chain pocket formed by residues Gly200, Ile202, Pro206, Phe239, His345, and Ile347 and thereby reduce potency. Analog 5a-14, with a terminal cyano group, retains some activity (MIC = 3.1 μM) due to the favorable interactions with the hydrophobic acyl chain pocket, whereas the terminal methoxy group of analog 5a-15 (MIC > 100 µM) may introduce clashes between lone pairs on the ether oxygen and proximal hydrophobic side chains (Gly200, Ile202, Pro206, and Phe239). Interestingly, 5a-12 and 5a-30, with an *n*-pentyl sulfonamide, offer increased hydrophobic interactions, presenting an explanation for the 2-fold increase in potency over DG167.

Our results validate KasA as a drug discovery target within M. tuberculosis with the potential to rapidly kill M. tuberculosis and perhaps shorten treatment in clinical TB, especially when used in combination with INH. Our results also support efforts to find other inhibitors along the mycolic acid biosynthesis pathway, as targeting multiple biosynthetic steps in cell wall biosynthesis may produce desirable features that could ultimately improve clinical TB treatment.

## MATERIALS AND METHODS

### Bacterial strains, culture conditions, primers, and plasmids.

M. tuberculosis strains were obtained from laboratory stocks. Clinical strains were obtained from a collection of clinical isolates from the Special Programme for Research and Training in Tropical Diseases (TDR) established by UNICEF/UNDP/World Bank/WHO Special Programs. All M. tuberculosis strains were grown at 37°C in Middlebrook 7H9 medium (Becton, Dickinson, Sparks, MD) enriched with 10% oleic acid-albumin-dextrose-catalase (OADC) (Becton, Dickinson) or 1× ADS (albumin [0.5 g/liter]-dextrose [0.2 g/liter]-sodium chloride [0.081 g/liter]) and Tween 80 (0.05% [wt/vol]) or tyloxapol (0.05% [wt/vol]) in liquid media. Middlebrook 7H10 agar (Becton, Dickinson) supplemented with 10% (vol/vol) OADC and 0.5% (vol/vol) glycerol was used to grow strains on solid media.

### Reporter screen for cell wall biosynthesis inhibitors.

A total of 168 compounds previously identified as having antitubercular activity in a whole-cell screen by GlaxoSmithKline (8) were tested for their ability to induce the *iniBAC* promoter (P_*iniBAC*_). The promoter screen used a BCG strain (BCG^S^ [pG4697-6]) harboring the *iniBAC* promoter sequence fused to a *lacZ* reporter on an integrative plasmid (pG4697-6) (10). BCG^S^ (pG4697-6) was grown to an optical density at 600 nm (OD_600_) of 0.2 to 0.3, 90 μl was dispensed into each well of 96-well deep well plates, and then 10 µl of each compound (final concentration, 10 µM) was added and the reaction mixture incubated at 37°C with shaking at 250 rpm. After incubation for 24 h, 100 µl of Lac Z buffer (60 mM Na_2_HPO_4_·7H_2_O, 40 mM NaH_2_PO_4_·H_2_O, 10 mM KCl, 1 mM MgSO_4_·7H_2_O, 50 mM β-mercaptoethanol) was added followed by addition of 5 µl of chloroform and 2 µl of 0.1% SDS, and the reaction mixture was then incubated for 5 min at room temperature (RT). Then, 40 µl of 4 mg/ml of LacZ substrate (2-nitrophenyl β-d-galactopyranoside; Sigma-Aldrich, St. Louis, MO) was added to each well and plates were incubated for 15 min. The reactions were terminated by adding 100 µl of 1 M aqueous sodium carbonate, and absorbance was read at 420 nm. Fold induction was determined by calculating the OD_420_ with compounds/OD_420_ of vehicle (dimethyl sulfoxide [DMSO]) controls. EMB and INH were used as positive controls.

### MIC and drug interaction.

MIC assays were performed in the 96-well format using the microdilution method (44). MIC assays were performed in 96-well plates using the microdilution alamarBlue (MABA) method. Briefly, the drugs were serially diluted in 50 µl of growth media (7H9-ADS) and supplemented 50-µl cultures (diluted 1:1,000) of M. tuberculosis grown to an OD_595_ of 0.2 to 0.3. After incubation for 7 days at 37°C, alamarBlue cell viability reagent (Thermo Fisher Scientific, Grand Island, NY, USA) was added, the cultures were incubated for another 24 h, and then the absorbance was read at 570 nm and normalized to 600 nM per the manufacturer’s instructions. A checkerboard analysis (45) was used to determine the synergy and antagonism of DG167 with INH. The fractional inhibitory concentration (FIC) was determined by dividing the MIC of the combination of drugs by the MIC of each drug measured independently. Summation fractional inhibitory index (ΣFIC) data were determined by adding the FICs of each drug tested. The activity levels of the compounds were defined as synergistic for ΣFIC values of ≤0.5, antagonistic for ΣFIC values of ≥4.0, and additive for ΣFIC values of >0.5 and <4.0 (45).

### Isolation of resistant mutants and whole-genome sequencing.

The DG167-resistant M. tuberculosis mutants were isolated by plating 10^6^ to 10^8^ M. tuberculosis cells onto 7H10 plates containing 1× to 32× DG167. Plates were screened for DG167-resistant colonies after 3 to 4 weeks at 37°C. The genomic DNA was isolated (46) and subjected to whole-genome sequencing and single nucleotide polymorphism (SNP) analysis (12).

### Cloning and purification of His-KasA.

The M. tuberculosis *kasA* gene was PCR amplified using AccuPrime SuperMix (Thermo Fisher Scientific) and primers *kasA*-NheI (5′-CGAGGCTTGAGGCCGAGCTAGCGTGAGTCAGCCTTC-3′) and *kasA*-HindIII (5′-CCCGCGATGTCAAGCTTCAGTAACG-3′). The *kasA* amplicon was cloned between NheI and HindIII restriction sites in plasmid pET28b, inserting an N-terminal His_6_ tag. The *kasA* gene, along with the His tag, was again PCR amplified using AccuPrime SuperMix and primers *kasA*-SR113-Inf-Fp (5′-AAAGGGAGTCCATATGGGCAGCAGCCATCATCAT-3′) and *kasA*-SR113-Inf-Fp (5′-GATAAGCTTCGAATTCTCAGTAACGCCCGAAGGC-3′) and cloned in an acetamide-inducible mycobacterial expression vector, pSR113 (47), between NdeI and EcoRI using an In-Fusion HD cloning kit (TaKaRa Bio USA). The resultant construct, pSR113-hisN-*kasA*, was transformed into Mycobacterium smegmatis mc^2^155. His-KasA was overexpressed in M. smegmatis grown in LB medium supplemented with 30 μg/ml kanamycin and 0.02% Tween 80 to an OD_600_ of 0.6 at 37°C. Expression was induced with 0.02% (wt/vol) acetamide for 36 h at 37°C. KasA was then purified using a modified version of a previously described protocol (28). Briefly, following expression, the bacterial cells were collected via centrifugation at 5,000 × *g* for 30 min and stored at −80°C. The cell pellet was resuspended in buffer A {500 mM NaCl, 20 mM CHES [2-(cyclohexylamino)ethanesulfonic acid] [pH 9.5]} accompanied by 20 μg/ml DNase. Cells were then lysed via the use of a French press at 15,000 lb/in^2^, and insoluble cell debris was separated via centrifugation at 25,000 × *g* for 45 min. Lysate supernatant was applied to His-60 Ni resin (Clontech) equilibrated in buffer A, and KasA was eluted with buffer A containing 200 mM imidazole. The eluted protein was then diluted to a final NaCl concentration of 50 mM using 20 mM CHES (pH 9.5) and loaded onto a MonoQ anion exchange column (GE Healthcare) equilibrated in 20 mM CHES (pH 9.5). KasA was eluted in a 50 to 1,000 mM NaCl gradient of 20 mM CHES (pH 9.5) over 20 column volumes (CV). Fractions containing KasA were pooled, concentrated using 10,000-molecular-weight-cutoff centrifugal filter units at 4,000 × *g*, and further purified by passage over a Superdex-200 16/70 column (GE Healthcare) equilibrated in buffer A. The final protein was then filtered through 0.22-μM-pore-size Costar spin filters and stored at 4°C. All protein concentrations were determined by UV spectroscopy at 280 nm using molar extinction coefficients experimentally derived by the method of Gill and von Hippel (48).

### Crystallization and data collection.

KasA crystals were produced by the vapor diffusion method at 20°C with 4.7 mg/ml of KasA in 2-μl hanging drops mixed 1:1 with mother liquor containing 200 mM NaCl with either 8% isopropanol and 1 mM Tris–(2-carboxyethyl)phosphine hydrochloride (TCEP HCl) or 14% isopropanol and 2 mM TCEP HCl. Crystals from the condition containing 8% isopropanol were used to determine the structure of KasA-DG167 and KasA-5a-30 (trideuteriomethyl analog of DG167), while crystals from the condition containing 14% isopropanol were used to determine the structure of apo-KasA. These conditions are similar but not identical to those previously used for crystallographic studies of KasA, which included the use of 10% isopropanol, 200 mM NaCl, 100 mM HEPES (pH 7.5), and 10 mM TCEP HCl (28). KasA-DG167 crystals were obtained by moving apo-KasA crystals from the hanging drops to 5 μl of soaking solutions containing 1 mM DG167, 8% isopropanol, 1 mM TCEP, 200 mM NaCl, and 1% DMSO for 1 h. After 1 h, the soaked crystal was placed in the same solution supplemented with 30% glycerol, immediately removed from the drop, and then flash-cooled in liquid nitrogen. KasA-5a-30 crystals were obtained in an identical manner by substituting the DG167 for 1 mM 5a-30. KasA-5g crystals were grown in a similar manner with the following modification: the 2-μl hanging drops contained 1 μl of mother liquor and 1 μl of purified 110 μM KasA and 1 mM 5g. The KasA-5g crystals selected for data collection were grown in 200 mM NaCl–2 mM TCEP HCl–4% isopropanol over the course of 7 days. These crystals were cryo-protected under their crystallization conditions with supplementation of 22% glycerol and 1 mM 5g.

X-ray diffraction data were collected using single crystals mounted in nylon loops that were then flash-cooled in liquid nitrogen before data collection in a stream of dry N_2_ at 100 K. Data sets for apo-KasA, KasA-DG167, and KasA-5a-30 were collected at Stanford Synchrotron Radiation Lightsource (SSRL) beamline 14-1 at 1.1808 Å with a MARmosaic 325 charge-coupled-device (CCD) detector. The KasA-5g data set was collected on beamline 9-2 at 0.88557 Å with a Dectris Pilatus 6M detector. X-ray data were processed using HKL2000 (49). The crystallographic phases were determined for apo-KasA by molecular replacement using Phaser (50) and the previously determined structure of apo-KasA (PDB code 2WGD) as a search model (28). The crystallographic phases were determined for KasA-DG167, KasA-5a-30, and KasA-5g by molecular replacement using Phaser (50) and the previously determined structure of KasA bound to TLM5 (PDB code 4C6U) (23). Models were generated using iterative cycles of model building in Coot (51) and refinement in phenix.refine (52). Initial refinement included simulated annealing as well as rigid body, individual atomic coordinate, and individual B-factor refinement. Later rounds of refinement employed individual atomic coordinate, individual B-factor, and TLS (Translation/Libration/Screw) refinement. TLS groups were selected using the TLSMD server (53). During the final rounds of refinement, the stereochemistry and ADP weights were optimized. DG167, 5a-30, 5g, isopropanol, glycerol, TCEP, ions, and water molecules were included only after the KasA models were complete. The following residues were omitted from the model (insufficient electron density was observed in flexible regions of the structures): apo-KasA (residues 1 to 25), KasA-DG167 (residues 1 to 25), and KasA-5a-30 (residues 1 to 25). Ramachandran statistics were calculated in Molprobity (54). Molecular graphics were produced with PyMOL (55).

### Microscale thermophoresis binding assays.

Prior to labeling, His-KasA was diluted from 100 μM in buffer A to 200 nM in buffer B (10 mM HEPES, 150 mM NaCl, pH 7.4). The diluted His-KasA was labeled using a Red-Tris-nitrilotriacetic acid (NTA) His tag labeling Kit (NanoTemper Technologies). Working stocks of labeled protein (50 nM) were made and consisted of buffer B supplemented with 0.1% poloxamer 407 (Pluronic F-127). Threefold titrations of 5a-2 and DG167 were made in DMSO, transferred by the use of a Labcyte 555 Echo instrument into separate 384-well polypropylene plates, and incubated with 50 nM working stock solutions of labeled protein in the dark for 30 min at room temperature. After incubation, the samples were transferred into Premium Coated Capillaries and read in a Monolith NT.115 Nano-Blue/Red instrument at room temperature using 60% light-emitting-diode (LED) power and 60% MST power for DG167 and 60% LED power and 40% MST power for 5a-2. Binding affinities were calculated using the thermophoresis with T jump evaluation strategy and a minimum of three experiments.

### Analysis of mycolic acids.

The mycolic acid methyl esters (MAMEs) and fatty acid methyl esters (FAMEs) (56) were analyzed as described previously (11). The compounds were added to 5 ml of M. tuberculosis cultures (OD_595_ of ∼0.3 to 0.4) and incubated at 37°C for 2 h, and 1 µCi/ml of [^14^C]acetate (56 mCi/mmol) was added to each culture, followed by incubation at 37°C for another 4 h. The ^14^C-labeled cells were pelleted by centrifugation, resuspended in 2 ml of tetra-*n*-butylammonium hydroxide (TBAH), and incubated overnight at 100°C to hydrolyze cell wall-bound lipids. The fatty acids were esterified by adding 4 ml CH_2_Cl_2_, 300 µl iodomethane, and 2 ml distilled water (dH_2_O) and mixing at room temperature for 1 h; then, the phases were separated by centrifugation, the upper aqueous phase was discarded, and the lower organic phase was washed twice with dH_2_O, dried, and resuspended in 3 ml of diethyl ether. Insoluble material was removed by centrifugation, the organic phase was dried, and lipids were resuspended in 200 µl CH_2_Cl_2_. Equal counts (20,000 cpm) were loaded on a silica gel 60 F254 thin-layer chromatography (TLC) plate and resolved using hexane/ethyl acetate (19:1 [vol/vol], 2 runs). The FAMEs and MAMEs were detected by phosphorimaging.

### Killing studies using CFU measurements.

M. tuberculosis cells (∼10^7^ CFU/ml) were treated with compounds and incubated at 37°C under shaking conditions, the samples were drawn at specific time points, and total viable counts were determined by dilution plating on 7H10-OADC-agar plates and counting CFU after 4 weeks of incubation at 37°C.

### Hu-Coates 100-day-old-culture model.

M. tuberculosis was grown in 10 ml of 7H9 medium supplemented with OADC and 0.025% Tween 80 in 25-ml screw cap Nunc tubes in a static incubator for 100 days. To disperse clumps, the cultures were sonicated using a low-intensity water bath sonicator (Branson 1800) by applying 3 pulses of 2 min of sonication and 1 min of rest followed by vortex mixing. The cidal activity of each compound and each compound combination against 100-day-old bacilli was determined by exposing each tube to the respective compounds for 5 days at 37°C followed by three washes with PBS to remove drug carryover and then measuring CFU by plating appropriate dilutions on 7H11-OADC- agar plates (35).

### RNA-seq analysis.

M. tuberculosis H37Rv was grown to an OD_595_ of ∼0.4 in tissue culture flasks (50 ml each), and the cultures were pooled. The cultures (10 ml) were redistributed into flasks containing each compound (10× MIC) or the compound combination or vehicle (DMSO) control. The final concentration of DMSO was kept constant in each flask. After 4 h of incubation at 37°C with shaking, the cultures were harvested by centrifugation and total RNA was extracted using TRIzol LS reagent (Thermo Fisher Scientific) and bead beating followed by extraction performed with an RNeasy minikit (Qiagen) as previously described (57). The integrity and purity of RNA were determined by the use of a bioanalyzer (model 2100; Agilent), rRNA was removed, and the cDNA library was prepared. The sequencing of the cDNA libraries was performed on an Illumina NextSeq 500 platform (Illumina, San Diego, CA) using the high-output (1 × 75 cycles) configuration. CLC Genomics Workbench, 9.0.1 version (Qiagen), was utilized for RNA-seq analysis. Demultiplexed fastq files from RNA-seq libraries were imported into the CLC software. Bases with low quality were trimmed, and reads were mapped to the reference genome, Mycobacterium tuberculosis H37Rv (NCBI reference sequence NC_000962.3). The aligned reads were obtained using the RNA-Seq Analysis Tool of the CLC Genomics Workbench. Values corresponding to the number of reads per kilobase per million (RPKM) were calculated for each gene to quantify absolute expression. Statistical analysis of differentially expressed genes was carried out with the Empirical analysis of Digital Gene Expression data tool in CLC Genomic Workbench. Data from replicates were averaged, and genes with a false-discovery-rate (FDR) adjusted *P* value of <0.05 and fold change at an absolute value of >2.0 are listed in Table S2 in the supplemental material. Raw transcriptomic data are available with GEO accession number GSE123132.

### Metabolomics.

One milliliter of M. tuberculosis H37Rv culture at an OD_595_ of 1.0 was transferred to a nitrocellulose filter, which was placed on 7H10 agar at 37°C for 5 days. On the day 5, the filter was moved to a 7H10 plate containing drug or vehicle and incubated at 37°C for 16 h. The filter was then plunged into 1 ml of acetonitrile-methanol-water (2:2:1 [vol/vol/vol]) and kept in a dry ice bath. The bacteria suspended in the buffer were transferred into 2-ml screw-cap tubes containing 0.1-mm-diameter zirconium beads and lysed by bead-beating on a Fastprep homogenizer employing a 30-s pulse at 6 m/s with intermittent cooling down for 1 min on ice. The insoluble fraction was pelleted by centrifugation at 4°C for 10 min and the soluble fraction frozen at −80°C until analysis. For liquid chromatography-mass spectrometry (LC-MS), samples were thawed and LC-MS and data analysis were performed as previously described (58–60). Data analysis was conducted with Agilent MassHunter Profinder Version B.06.00. Peaks in the high-performance liquid chromatography (HPLC) trace of the appropriate M. tuberculosis sample were searched for drug and its metabolites and for glutamate via their exact mass. Their respective ion counts were quantified by integration of the peak for each replicate. The reported ion counts for drug and metabolites under each condition were calculated by normalizing for each replicate the compound’s ion count by the corresponding ion count for glutamate and then averaging the data from the three replicates. Plotted ion count values represent averages of results from experimental triplicates ± standard error (SE).

### Mouse pharmacokinetic studies.

All animal experiments were approved by the Institutional Animal Care and Use Committee of the New Jersey Medical School, Rutgers University, and were conducted in compliance with their guidelines. Female BALB/c mice (23 to 29 g) were weighed and treated via oral gavage with a single dose of DG167 (100 mg/kg of body weight) formulated in 0.5% carboxymethyl cellulose (CMC)–0.5% Tween 80. Sequential bleeds were collected at 0.25, 0.5, 1, 3, 5, and 8 h postdose via the tail snip method. Blood (50 µl) was collected in capillary microvette EDTA blood tubes and kept on ice prior to centrifugation at 1,500 × *g* for 5 min. The supernatant (plasma) was transferred into a 96-well plate and stored at −80°C. In a dose escalation study, mice were dosed with 50, 100, 250, or 500 mg/kg DG167, and blood was similarly sampled and processed.

### Quantitative analysis.

DG167 levels in plasma were measured by LC-tandem MS (LC-MS/MS) in positive electrospray ionization (ESI^+^) mode on a Sciex Qtrap 4000 triple-quadrupole MS system combined with an Agilent 1260 HPLC using Analyst software. Chromatography was performed with an Agilent Zorbax SB-C8 column (2.1 by 30 mm; particle size, 3.5 µm) using a reverse-phase gradient elution. A mixture of 0.1% formic acid–Milli-Q deionized water was used for the aqueous mobile phase and 0.1% formic acid–acetonitrile (ACN) for the organic mobile phase. Multiple-reaction monitoring (MRM) of parent/daughter transitions in positive electrospray ionization (ESI^+^) mode was used to quantify DG167. A DMSO stock of DG167 was serial diluted in blank K_2_EDTA (dipotassium ethylenediaminetetraacetic acid) plasma (Bioreclammation) to create standard curves and quality control samples. DG167 was extracted by combining 20 µl of spiked plasma or study samples and 200 µl of acetonitrile/methanol (50/50) protein precipitation solvent containing 20 ng/ml verapamil internal standard (IS). Extracts were subjected to vortex mixing for 5 min and centrifuged at 4,000 rpm for 5 min. The supernatants were analyzed by LC-MS. Verapamil IS was sourced from Sigma-Aldrich. MRM transition values of 268.1 and 146 were used for DG167 and MRM transition values of 455.4 and 165.2 for verapamil. The sample analysis results were accepted if the concentrations of the quality control samples were within 20% of the nominal concentration.

### Drug tolerability.

Five mice were dosed orally daily for 5 days with DG167 (100 mg/kg) formulated in 0.5% CMC–0.5% Tween 80–INH (25 mg/kg) in water. Prior to dosing, DG167 and INH were mixed (1:1) and subjected to vortex mixing. The mice were weighed and observed daily. Their behavior, drinking and feeding patterns, and feces characteristics were monitored and recorded. Upon necropsy, liver, gallbladder, kidney, and spleen pathology was observed as well.

### Intracellular inhibition assay.

J774A.1 cells obtained from the American Type Culture Collection (ATCC, Manassas, VA) (TIB-67) were cultured in Dulbecco’s modified Eagle’s medium (DMEM; Sigma) supplemented with 10% fetal bovine serum (Sigma), and 2.5 × 10^4^ Please cells/well were seeded into white 96-well clear flat-bottom white tissue culture plates 24 h prior to infection. M. tuberculosis (mc^2^ 6206 –pan –leu) expressing luciferase was grown to mid-log phase, washed with Dulbecco's PBS (DPBS; Sigma), and diluted in DMEM supplemented with 10% fetal bovine serum, pantothenic acid, and leucine. The bacterial suspension was added to macrophages at multiplicity of infection (MOI) of 1. After 4 h of incubation at 37°C in 5% CO_2_, macrophages were treated with 50 µg/ml gentamicin for 1 h and washed twice to remove extracellular bacteria. Finally, 100 µl of drug diluted in DMEM supplemented with 10% fetal bovine serum, pantothenic acid, and leucine was added to each well. Inhibition of intracellular survival and growth was determined by CFU counting and by measuring luciferase activity with a luminometer 48 h after drug was added to the plates.

### Mouse efficacy.

BALB/c mice (9-week-old females; weight range, 18 to 20 g) were infected with an inoculum of M. tuberculosis H37Rv mixed with 5 ml of phosphate-buffered saline (PBS) (3 × 10^6^ CFU/ml) using a Glas-Col whole-body aerosol unit. This resulted in lung implantation of 1.09 log_10_ CFU per mouse. Groups of 5 mice were sacrificed by cervical dislocation at the start of treatment (2 week postinfection) and after receiving DG167 at 100 mg/kg, INH at 25 mg/kg, the drug combination (DG167 at 100 mg/kg plus INH at 25 mg/kg), or the vehicle only daily for 3 days, 1 week, or 2 weeks. Whole lungs were homogenized in 5 ml of PBS containing 0.05% Tween 80. CFU counts were determined by plating serial dilutions of homogenates onto Middlebrook 7H11 agar with OADC. Colonies were counted after at least 21 days of incubation at 37°C. The data were plotted, and statistical analysis was performed using GraphPad Prism 7. Significance was determined using the Kruskal-Wallis test and one-way analysis of variance (ANOVA) for multiple comparisons to generate *P* values.

### Synthesis of DG167 and its analogs.

All reagents were purchased from commercial suppliers and used without further purification unless noted otherwise. All chemical reactions occurring solely in an organic solvent were carried out under conditions of an inert atmosphere of argon or nitrogen. Analytical TLC was performed with Merck silica gel 60 F_254_ plates. Silica gel column chromatography was conducted with Teledyne Isco CombiFlash Companion or Rf+ systems. ^1^H nuclear magnetic resonance (NMR) spectra were acquired on Bruker instruments (500 MHz) and are listed in parts per million downfield from tetramethylsilane (TMS). LC-MS was performed on an Agilent 1260 HPLC system coupled to an Agilent 6120 MS. All synthesized compounds were at least 95% pure as judged by their HPLC trace at 250 nm and were characterized by the expected parent ion(s) in the MS.

### Synthetic chemistry.

All reagents were purchased from commercial suppliers and used without further purification unless noted otherwise. All chemical reactions occurring solely in an organic solvent were carried out under an inert atmosphere of argon or nitrogen. Analytical TLC was performed with Merck silica gel 60 F_254_ plates. Silica gel column chromatography was conducted with Teledyne Isco CombiFlash Companion or Rf+ systems. ^1^H NMR spectra were acquired on Bruker 500 MHz instruments and are listed in parts per million downfield from TMS. LC-MS was performed on an Agilent 1260 HPLC system coupled to an Agilent 6120 mass spectrometer. All synthesized compounds were at least 95% pure as judged by their HPLC trace at 250 nm and were characterized by the expected parent ion(s) in the MS.

### Synthesis of 1-methyl-6-nitro-1*H*-indazole (2a).

NaH (2.03 g, 50.7 mmol) was added in four portions to a vigorously stirred, ice-cold solution of 6-nitro-1*H*-indazole (4.14 g, 25.4 mmol) in dimethylformamide (100 ml). The reaction mixture was maintained at 0°C for 30 min. Iodomethane (1.74 ml, 27.9 mmol) was added dropwise to the reaction mixture, and the reaction mixture was stirred for 16 h at room temperature. The reaction mixture was quenched with water and diluted with ethyl acetate. The reaction mixture was transferred to a separatory funnel, washed with water three times, and dried over anhydrous magnesium sulfate. The reaction mixture was concentrated on a rotary evaporator, and 1-methyl-6-nitro-1*H*-indazole was separated from 2-methyl-6-nitro-2*H*-indazole by flash chromatography on silica using ethyl acetate as the eluent to afford the product as a yellow solid (2.54 g, 56.4% yield) as follows: ^1^H NMR (600 MHz, DMSO) δ 8.73 (s, 1H), 8.29 (s, 1H), 8.01 (d, *J *=* *8.79 Hz, 1H), 7.95 (dd, *J *=* *1.47, 8.79 Hz, 1H), 4.19 (s, 3H).

The same basic procedure was followed for the synthesis of 1-ethyl-6-nitro-1*H*-indazole (2b), 6-nitro-1-propyl-1*H*-indazole (2c), 1-benzyl-6-nitro-1*H*-indazole (2d), and 1-(methyl-d3)-6-nitro-1*H*-indazole (2e).

### Synthesis of 1-isopropyl-6-nitro-1*H*-indazole (2f).

A solution of 6-nitro-1*H*-indazole (378 mg, 2.32 mmol), isopropyl iodide (277.5 µl, 2.780 mmol), copper (I) iodide (22 mg, 0.12 mmol), potassium phosphate (985 mg, 4.64 mmol), *N*,*N*-dimethylethylenediamine (25.3 µl, 0.232 mmol), and dimethylformamide (1.2 ml) was stirred at 110°C for 72 h. After completion of the reaction, the reaction mixture was filtered through a pad of Celite and the filtrate was diluted with ethyl acetate, transferred to a separatory funnel, washed with water three times, and dried over anhydrous magnesium sulfate. The reaction mixture was concentrated on a rotary evaporator, and 1-isopropyl-6-nitro-1*H*-indazole was separated from 2-isopropyl-6-nitro-2*H*-indazole by flash chromatography on silica using ethyl acetate as the eluent to afford the product as a yellow solid (199 mg, 56.4% yield) as follows: ^1^H NMR (500 MHz, CDCl_3_) δ 7.97 (s, 1H), 7.67 (d, *J *=* *8.54 Hz, 1H), 7.43 (s, 1H), 6.86 (dd, *J *=* *1.8, 8.5 Hz, 1H), 6.63 (s, 1H), 4.80 (td, *J *=* *6.6, 13.3 Hz, 1H), 3.03 to 3.20 (m, 2H), 1.72 to 1.92 (m, 2H), 1.58 (d, *J *=* *6.4 Hz, 6 + 1 H from H_2_O), 1.31 to 1.51 (m, 2H), 0.90 (t, *J *=* *7.3 Hz, 3H).

1-Phenyl-6-nitro-1*H*-indazole (2g) was synthesized following the same basic procedure.

### Synthesis of 1-methyl-1*H*-indazol-6-amine (4a).

To a solution of 1-methyl-6-nitro-1*H*-indazole (2a) (2.5 g, 14.2 mmol) in ethanol (150 ml) was added ammonium formate (7 g) and 10 wt% Pd/C (1 g). The mixture maintained under nitrogen was stirred at room temperature for 3 h. After completion of the reaction, the Pd/C catalyst and excess ammonium formate were removed via filtration of the crude reaction mixture through a pad of Celite. The filtrate was concentrated on the rotary evaporator to remove ethanol. The crude material was purified by flash chromatography on a silica gel to obtain 1-methyl-1*H*-indazol-6-amine (4a) as a light pink solid (1.61 g, 77% yield) as follows: ^1^H NMR (500 MHz, CDCl_3_) δ 7.80 (s, 1H), 7.48 (d, *J *=* *8.54 Hz, 1H), 6.57 (d, *J *=* *8.54 Hz, 1H), 6.53 (s, 1H), 3.94 (s, 3H), 3.88 (br s, 2H).

The same basic procedure was followed for the synthesis of 1-ethyl-1*H*-indazol-6-amine (**4b),** 1-propyl-1*H*-indazol-6-amine (**4c**), 1-benzyl-1*H*-indazol-6-amine (**4d**), 1-(methyl-d3)-1*H*-indazol-6-amine (**4e**), 1-isoproyl-1*H*-indazol-6-amine (**4f**), 1-phenyl-1*H*-indazol-6-amine (**4g**) and 3-methyl-5-nitro-1*H*-indazole (**4h**).

### *N*-(1-Methyl-1*H*-indazol-6-yl)butane-1-sulfonamide (5a-1).

To a solution of **4a** (464 mg, 3.15 mmol) in pyridine (20 ml) was added *n*-butyl sulfonyl chloride (450 µl, 3.47 mmol) and the reaction mixture was stirred at room temperature for 16 h. After completion of the reaction, the reaction mixture was diluted with ethyl acetate and transferred to a separatory funnel. The organic layer was washed with saturated aqueous sodium bicarbonate solution followed by water and brine. The organic layer was dried over anhydrous magnesium sulfate, filtered, and concentrated. The crude product was purified by flash chromatography on a silica gel (gradient, 30% to 70% ethyl acetate/hexanes) to obtain the product as a white solid (700 mg, 83% yield) as follows: ^1^H NMR (500 MHz, CDCl_3_) δ 7.94 (s, 1 H), 7.67 (d, *J *=* *8.5 Hz, 1 H), 7.39 (s, 1 H), 7.11 (br. s., 1 H), 6.91 (dd, *J *=* *1.8, 8.5 Hz, 1 H), 4.05 (s, 3 H), 3.17 to 3.09 (m, 2 H), 1.89 to 1.77 (m, 2 H), 1.45 to 1.35 (m, 2 H), 0.88 (t, *J *=* *7.5 Hz, 3 H).

The same basic procedure was followed for the synthesis of 5a-2 to 5a-32 and 5e.

### *N*-(1*H*-Indazol-6-yl)butane-1-sulfonamide (5a-2).

^1^H NMR (500 MHz, CDCl_3_) δ 8.06 (s, 1H), 7.49 (d, *J *=* *8.54 Hz, 1H), 6.70 (dd, *J *=* *1.83, 8.54 Hz, 1H), 4.07 (br s, 1H), 3.31 to 3.41 (m, 2 H), 1.66 (td, *J *=* *7.67, 15.49 Hz, 2 H), 1.36 (qd, *J *=* *7.44, 14.92 Hz, 2 H), 0.86 (t, *J *=* *7.32 Hz, 3 H).

### *N*-(1-Ethyl-1*H*-indazol-6-yl)butane-1-sulfonamide (5a-3).

^1^H NMR (500 MHz, CDCl_3_) δ 7.90 to 8.00 (m, 1H), 7.67 (d, *J *=* *8.5 Hz, 1H), 7.41 (s, 1H), 6.87 (d, *J *=* *8.5 Hz, 1H), 6.77 (s, 1H), 4.41 (q, *J *=* *7.3 Hz, 2H), 3.07 to 3.18 (m, 2H), 1.82 (quin, *J *=* *7.7 Hz, 2H), 1.51 (t, *J *=* *7.3 Hz, 3H), 1.41 (qd, *J *=* *7.3, 14.9 Hz, 2H), 0.89 (t, *J *=* *7.3 Hz, 3H).

### *N*-(1-Propyl-1*H*-indazol-6-yl)butane-1-sulfonamide (5a-4).

^1^H NMR (500 MHz, CDCl_3_) δ 7.95 (s, 1H), 7.67 (d, *J *=* *8.54 Hz, 1H), 7.40 (s, 1H), 6.85 to 6.96 (m, 2H), 4.31 (t, *J *=* *7.02 Hz, 2H), 3.06 to 3.16 (m, 2H), 1.89 to 1.99 (m, 2H), 1.76 to 1.87 (m, 2H), 1.34 to 1.45 (m, 2H), 0.82 to 0.96 (m, 6H).

### *N*-(1-Isoproply-1*H*-indazol-6-yl)butane-1-sulfonamide (5a-5).

^1^H NMR (500 MHz, CDCl_3_) δ 7.97 (s, 1H), 7.67 (d, *J *=* *8.54 Hz, 1H), 7.43 (s, 1H), 6.86 (dd, *J *=* *1.83, 8.54 Hz, 1H), 6.63 (s, 1H), 4.80 (td, *J *=* *6.60, 13.35 Hz, 1H), 3.03 to 3.20 (m, 2H), 1.72 to 1.92 (m, 2H), 1.58 (d, *J *=* *6.41 Hz, 6× H + 1 H from H_2_O), 1.31 to 1.51 (m, 2H), 0.90 (t, *J *=* *7.32 Hz, 3H).

### *N*-(1-Phenyl-1*H*-indazol-6-yl)butane-1-sulfonamide (5a-6).

^1^H NMR (500 MHz, CDCl_3_) δ 8.17 (s, 1H), 7.75 (d, *J *=* *8.55 Hz, 1H), 7.65 to 7.73 (m, 3H), 7.55 (t, *J *=* *7.93 Hz, 2H), 7.33 to 7.42 (m, 1H), 7.02 (dd, *J *=* *1.68, 8.70 Hz, 1H), 6.70 (s, 1H), 3.06 to 3.17 (m, 2H), 1.76 to 1.87 (m, 2H), 1.34 to 1.45 (m, 2H), 0.89 (t, *J *=* *7.32 Hz, 3H).

### *N*-(1-Benzyl-1*H*-indazol-6-yl)butane-1-sulfonamide (5a-7).

^1^H NMR (500 MHz, CDCl_3_) δ 8.00 (s, 1H), 7.67 (d, *J *=* *8.54 Hz, 1H), 7.14 to 7.43 (m, 5H), 6.87 (d, *J *=* *8.54 Hz, 1H), 6.60 (s, 1H), 5.57 (s, 2H), 2.92 to 3.11 (m, 2H), 1.74 (t, *J *=* *7.63 Hz, 2H), 1.28 to 1.43 (m, 2H), 0.85 (t, *J *=* *7.32 Hz, 3H).

### *N*-(1-Methyl-1*H*-indazol-6-yl)methanesulfonamide (5a-8).

^1^H NMR (500 MHz, CD_3_OD) δ 7.94 (s, 1H), 7.70 (d, *J *=* *8.5 Hz, 1H), 7.41 (s, 1H), 7.03 (d, *J *=* *8.5 Hz, 1H), 4.02 (s, 3H), 3.00 (s, 3H).

### *N*-(1-Methyl-1*H*-indazol-6-yl)ethanesulfonamide (5a-9).

^1^H NMR (500 MHz, CDCl_3_) δ 7.94 (br s, 1H), 7.67 (d, *J *=* *7.93 Hz, 1H), 7.39 (br s, 1H), 6.96 (br s, 1H), 6.91 (d, *J *=* *7.63 Hz, 1H), 4.05 (br s, 3H), 3.04 to 3.27 (m, 2H), 1.29 to 1.47 (m, 3H).

### *N*-(1-Methyl-1*H*-indazol-6-yl)propane-1-sulfonamide (5a-10).

^1^H NMR (500 MHz, CDCl_3_) δ 7.94 (s, 1H), 7.67 (d, *J *=* *8.5 Hz, 1H), 7.39 (s, 1H), 6.86 (d, *J *=* *8.5 Hz, 1H), 6.61 (s, 1H), 4.06 (s, 3H), 3.06 to 3.15 (m, 2H), 1.88 (qd, *J *=* *7.5, 15.3 Hz, 2H), 1.02 (t, *J *=* *7.3 Hz, 3H).

### *N*-(1-Methyl-1*H*-indazol-6-yl)propane-2-sulfonamide (5a-11).

^1^H NMR (500 MHz, acetone) δ 8.75 (br s, 1H), 7.90 (s, 1H), 7.70 (d, *J *=* *8.54 Hz, 1H), 7.51 (s, 1H), 7.16 (dd, *J *=* *1.68, 8.70 Hz, 1H), 4.01 (s, 3H), 3.37 (td, *J *=* *6.75, 13.66 Hz, 1H), 1.33 (d, *J *=* *7.02 Hz, 6H).

### *N*-(1-Methyl-1*H*-indazol-6-yl)pentane-1-sulfonamide (5a-12).

^1^H NMR (500 MHz, CDCl_3_) δ 7.94 (s, 1H), 7.67 (d, *J *=* *8.5 Hz, 1H), 7.39 (s, 1H), 6.88 (d, *J *=* *8.5 Hz, 1H), 6.77 (br s, 1H), 4.06 (s, 3H), 3.08 to 3.15 (m, 2H), 1.84 (quint, *J *=* *7.7 Hz, 2H), 1.23 to 1.40 (m, 4H), 0.86 (t, *J *=* *7.2 Hz, 3H).

### *N*-(1-Methyl-1*H*-indazol-6-yl)hexane-1-sulfonamide (5a-13).

^1^H NMR (500 MHz, CDCl_3_) δ 7.95 (s, 1H), 7.67 (d, *J *=* *8.54 Hz, 1H), 7.39 (s, 1H), 6.88 (d, *J *=* *8.24 Hz, 1H), 6.81 (br s, 1H), 4.06 (s, 3H), 3.06 to 3.16 (m, 2H), 1.78 to 1.89 (m, 2H), 1.31 to 1.42 (m, 2H), 1.24 (br s, 2H^+^ grease), 0.81 to 0.87 (m, 3H).

### 4-Cyano-*N*-(1-methyl-1*H*-indazol-6-yl)butane-1-sulfonamide (5a-14).

^1^H NMR (500 MHz, CDCl_3_) δ 7.95 (s, 1H), 7.69 (d, *J *=* *8.5 Hz, 1H), 7.39 (s, 1H), 6.91 (d, *J *=* *8.2 Hz, 1H), 6.80 (s, 1H), 4.06 (s, 3H), 3.17 (t, *J *=* *7.3 Hz, 2H), 2.38 (t, *J *=* *6.9 Hz, 2H), 2.02 (quin, *J *=* *7.5 Hz, 2H), 1.75 to 1.86 (m, 2H).

### 4-Methoxy-*N*-(1-methyl-1*H*-indazol-6-yl)butane-1-sulfonamide (5a-15).

^1^H NMR (500 MHz, CDCl_3_) δ 7.94 (s, 1H), 7.67 (d, *J *=* *8.5 Hz, 1H), 7.39 (s, 1H), 6.88 (d, *J *=* *8.2 Hz, 1H), 6.84 (br s, 1H), 4.06 (s, 3H), 3.34 (t, *J *=* *5.5 Hz, 2H), 3.26 (s, 3H), 3.17 (t, *J *=* *7.5 Hz, 2H), 1.94 (quin, *J *=* *7.4 Hz, 2H), 1.59 to 1.68 (m, 2H).

### 5-Methoxy-3-methyl-*N*-(1-methyl-1*H*-indazol-6-yl)pentane-1-sulfonamide (5a-16).

^1^H NMR (500 MHz, CDCl_3_) δ 7.94 (s, 1H), 7.66 (d, *J *=* *8.54 Hz, 1H), 7.39 (s, 1H), 7.19 (br s, 1H), 6.91 (dd, *J *=* *1.83, 8.54 Hz, 1H), 4.05 (s, 3H), 3.29 to 3.40 (m, 2H), 3.25 (s, 3H), 3.07 to 3.20 (m, 2H), 1.89 (ddd, *J *=* *5.49, 7.78, 10.83 Hz, 1H), 1.62 to 1.73 (m, 2H), 1.47 to 1.57 (m, 1H), 1.31 to 1.42 (m, 1H), 0.85 (d, *J *=* *6.41 Hz, 3H).

### *N*-(1-Methyl-1*H*-indazol-6-yl)hexane-3-sulfonamide (5a-17).

^1^H NMR (500 MHz, CDCl_3_) δ 7.93 (s, 1H), 7.66 (d, *J *=* *8.5 Hz, 1H), 7.39 (s, 1H), 6.85 (dd, *J *=* *1.83, 8.5 Hz, 1H), 6.57 (br s, 1H), 4.05 (s, 3H), 3.02 (quin, *J *=* *5.8 Hz, 1H), 1.74 to 1.98 (m, 4H), 1.45 to 1.57 (m, 1H), 1.33 to 1.45 (m, 1H), 1.03 (t, *J *=* *7.5 Hz, 3H), 0.89 (t, *J *=* *7.3 Hz, 3H).

### *N*-(1-Methyl-1*H*-indazol-6-yl)pentane-2-sulfonamide (5a-18).

^1^H NMR (500 MHz, CDCl_3_) δ 7.93 (s, 1H), 7.66 (d, *J *=* *8.5 Hz, 1H), 7.39 (s, 1H), 6.89 (d, *J *=* *8.5 Hz, 1H), 6.78 (s, 1H), 4.05 (s, 3H), 3.12 to 3.24 (m, 1H), 1.92 to 2.06 (m, 1H), 1.56 to 1.69 (m, 1H), 1.46 to 1.56 (m, 1H), 1.36 to 1.42 (m, 3H), 1.25 to 1.35 (m, 1H), 0.89 (t, *J *=* *7.3 Hz, 3H).

### 4-Methyl-*N*-(1-methyl-1*H*-indazol-6-yl)pentane-1-sulfonamide (5a-19).

^1^H NMR (500 MHz, CDCl_3_) δ 7.94 (s, 1H), 7.67 (d, *J *=* *8.54 Hz, 1H), 7.39 (s, 1H), 6.87 (dd, *J *=* *1.68, 8.70 Hz, 1H), 6.76 (br s, 1H), 4.06 (s, 3H), 3.01 to 3.14 (m, 2H), 1.76 to 1.90 (m, 2H), 1.51 (quind, *J *=* *6.64, 13.41 Hz, 1H), 1.22 to 1.29 (m, 2H), 0.85 (d, *J *=* *6.41 Hz, 6H).

### 4-Methyl-*N*-(1-methyl-1*H*-indazol-6-yl)benzenesulfonamide (5a-20).

^1^H NMR (500 MHz, CD_3_OD) δ 7.87 (br s, 1H), 7.67 (d, *J *=* *6.4 Hz, 2H), 7.56 (d, *J *=* *8.5 Hz, 1H), 7.23 to 7.31 (m, 3H), 6.86 (d, *J *=* *8.5 Hz, 1H), 3.95 (br s, 3H), 2.34 (br s, 3H).

### *N*-(1-Methyl-1*H*-indazol-6-yl)-1-phenylmethanesulfonamide (5a-21).

^1^H NMR (500 MHz, CDCl_3_) δ 7.93 (s, 1H), 7.62 to 7.73 (m, 1H), 7.27 to 7.41 (m, 5H), 6.72 (d, *J *=* *8.5 Hz, 1H), 6.57 (br s, 1H), 4.38 (s, 2H), 4.03 (s, 3H).

### *N*-(1-Methyl-1*H*-indazol-6-yl)-2-phenylethane-1-sulfonamide (5a-22).

^1^H NMR (500 MHz, CDCl_3_) δ 7.93 (s, 1H), 7.62 (d, *J *=* *8.5 Hz, 1H), 7.26 to 7.33 (m, 3H), 7.20 (s, 1H), 7.15 (d, *J *=* *7.3 Hz, 2H), 6.65 (d, *J *=* *8.5 Hz, 1H), 6.42 (s, 1H), 4.04 (s, 3H), 3.39 (t, *J *=* *7.6 Hz, 2H), 3.16 (t, *J *=* *7.6 Hz, 2H).

### *N*-(1-Methyl-1*H*-indazol-6-yl)cyclopropanesulfonamide (5a-23).

^1^H NMR (500 MHz, CDCl_3_) δ 7.95 (s, 1H), 7.67 (d, *J *=* *8.5 Hz, 1H), 7.41 (s, 1H), 6.95 (dd, *J *=* *1.9, 8.4 Hz, 1H), 6.60 (s, 1H), 4.06 (s, 3H), 2.46 to 2.59 (m, 1H), 1.16 to 1.23 (m, 2H), 0.91 to 1.01 (m, 2H).

### *N*-(1-Methyl-1*H*-indazol-6-yl)cyclobutanesulfonamide (5a-24).

^1^H NMR (500 MHz, CDCl_3_) δ 7.93 (s, 1H), 7.65 (d, *J *=* *8.54 Hz, 1H), 7.37 (s, 1H), 6.86 (d, *J *=* *8.55 Hz, 1H), 6.75 (br s, 1H), 4.05 (s, 3H), 3.94 (quin, *J *=* *8.32 Hz, 1H), 2.52 to 2.63 (m, 2H), 2.20 to 2.30 (m, 2H), 1.91 to 2.05 (m, 2H).

### *N*-(1-Methyl-1*H*-indazol-6-yl)cyclopentanesulfonamide (5a-25).

^1^H NMR (500 MHz, CDCl_3_) δ 7.93 (s, 1H), 7.66 (d, *J *=* *8.24 Hz, 1H), 7.41 (s, 1H), 6.87 (d, *J *=* *8.54 Hz, 1H), 6.54 (s, 1H), 4.05 (s, 3H), 3.50 to 3.62 (m, 1H), 1.93 to 2.14 (m, 5H), 1.78 to 1.87 (m, 2H), 1.58 to 1.64 (m, 2H).

### *N*-(1-Methyl-1*H*-indazol-6-yl)cyclohexanesulfonamide (5a-26).

^1^H NMR (500 MHz, CDCl_3_) δ 7.93 (s, 1H), 7.66 (d, *J *=* *8.54 Hz, 1H), 7.40 (s, 1H), 6.86 (d, *J *=* *8.54 Hz, 1H), 6.52 (s, 1H), 4.05 (s, 3H), 2.97 to 3.11 (m, 1H), 2.17 (d, *J *=* *12.82 Hz, 2H), 1.82 to 1.91 (m, 2H), 1.58 to 1.70 (m, 3H), 1.14 to 1.23 (m, 3H).

### *N*-(1-Methyl-1*H*-indazol-6-yl)-4-(trifluoromethyl)cyclohexane-1-sulfonamide (5a-27).

^1^H NMR (500 MHz, CDCl_3_) δ 7.94 (s, 1H), 7.67 (d, *J *=* *8.54 Hz, 1H), 7.39 (s, 1H), 6.87 (dd, *J *=* *1.83, 8.54 Hz, 1H), 6.65 (s, 1H), 4.05 (s, 3H), 3.22 (quin, *J *=* *5.57 Hz, 1H), 2.20 to 2.27 (m, 2H), 2.05 to 2.18 (m, 3H), 1.82 to 1.93 (m, 2H), 1.67 to 1.75 (m, 2H).

### *N*-(1-Methyl-1*H*-indazol-6-yl)piperidine-1-sulfonamide (5a-28).

^1^H NMR (500 MHz, CDCl_3_) δ 7.92 (s, 1H), 7.63 (d, *J *=* *8.54 Hz, 1H), 7.30 (s, 1H), 7.01 (br s, 1H), 6.90 (dd, *J *=* *1.53, 8.54 Hz, 1H), 4.04 (s, 3H), 3.21 to 3.31 (m, 4H), 1.50 to 1.57 (m, 4H), 1.43 to 1.50 (m, 2H).

### 1-Cyclohexyl-*N*-(1-methyl-1*H*-indazol-6-yl)methanesulfonamide (5a-29).

^1^H NMR (500 MHz, CDCl_3_) δ 7.94 (s, 1H), 7.67 (d, *J *=* *8.24 Hz, 1H), 7.37 (s, 1H), 6.87 (d, *J *=* *8.54 Hz, 1H), 6.81 (s, 1H), 4.05 (s, 3H), 3.01 (d, *J *=* *6.10 Hz, 2H), 1.99 to 2.13 (m, 1H), 1.87 to 1.99 (m, *J *=* *12.50 Hz, 2H), 1.64 to 1.75 (m, *J *=* *13.40 Hz, 2H), 1.21 to 1.36 (m, 2H), 0.98 to 1.21 (m, 3H).

### *N*-(1-(Methyl-d3)-1*H*-indazol-6-yl)butane-1-sulfonamide (5a-30).

^1^H NMR (500 MHz, CDCl_3_) δ 7.95 (s, 1H), 7.68 (d, *J *=* *8.5 Hz, 1H), 7.37 to 7.44 (m, 1H), 6.89 (dd, *J *=* *2.14, 8.5 Hz, 1H), 6.80 (br s, 1H), 3.10 to 3.17 (m, 2H), 1.78 to 1.87 (m, 2H), 1.41 (qd, *J *=* *7.5, 14.9 Hz, 2H), 0.90 (t, *J *=* *7.3 Hz, 3H).

### *N*-(1-(Methyl-d3)-1*H*-indazol-6-yl)pentane-1-sulfonamide (5a-31).

^1^H NMR (500 MHz, CDCl_3_) δ 7.95 (s, 1H), 7.67 (d, *J *=* *8.5 Hz, 1H), 7.38 (s, 1H), 6.87 (d, *J *=* *8.5 Hz, 1H), 6.70 (s, 1H), 3.06 to 3.17 (m, 2H), 1.84 (quint, *J *=* *7.5 Hz, 2H), 1.22 to 1.42 (m, 4H), 0.86 (t, *J *=* *7.0 Hz, 3H).

### 4-Methoxy-*N*-(1-(methyl-d3)-1*H*-indazol-6-yl)butane-1-sulfonamide (5a-32).

^1^H NMR (500 MHz, CDCl_3_) δ 7.94 (s, 1H), 7.67 (d, *J *=* *8.54 Hz, 1H), 7.38 (s, 1H), 6.87 (d, *J *=* *8.54 Hz, 1H), 6.70 (br s, 1H), 3.35 (t, *J *=* *5.80 Hz, 2H), 3.26 (s, 3H), 3.17 (t, *J *=* *7.48 Hz, 2H), 1.87 to 2.02 (m, 2H), 1.53 to 1.72 (m, 2H plus 1 H from H_2_O).

### *N*-(3-Methyl-1*H*-indazol-5-yl)butane-1-sulfonamide (5e).

^1^H NMR (500 MHz, CDCl_3_) δ 7.60 (d, *J *=* *1.53 Hz, 1H), 7.43 (d, *J *=* *8.85 Hz, 1H), 7.28 (d, *J *=* *1.83 Hz, 1H), 6.55 (s, 1H), 3.03 to 3.09 (m, 2H), 2.59 (s, 3H), 1.78 to 1.89 (m, 2H), 1.43 (sxt, *J *=* *7.45 Hz, 2H), 0.91 (t, *J *=* *7.32 Hz, 3H).

### 1-Butyl-3-(1-methyl-1*H*-indazol-6-yl)thiourea (5b-1).

*n*-Butylisothiocyanate (25 µl, 0.2 mmol) was added to a solution of 4a (27 mg, 0.183 mmol) in pyridine (1 ml), and the reaction mixture was stirred at room temperature for 14 h. After completion of the reaction, the reaction mixture was diluted with ethyl acetate, transferred to a separatory funnel, and washed with water, saturated sodium bicarbonate solution, and brine. The organic layer was dried over anhydrous magnesium sulfate and concentrated via rotary evaporator. The crude product was purified by flash chromatography on silica gel (0% to 70% ethyl acetate/hexanes) to obtain the product as a white solid (34.6 mg, 72% yield) as follows: ^1^H NMR (500 MHz, CDCl_3_) δ 8.00 (s, 1H), 7.77 (d, *J *=* *8.2 Hz, 2H), 6.98 (dd, *J *=* *1.2, 8.5 Hz, 1H), 6.05 (br s, 1H), 4.06 (s, 3H), 3.60 to 3.69 (m, 2H), 1.50 to 1.60 (m, 2H), 1.33 (qd, *J *=* *7.4, 15.0 Hz, 2H), 0.92 (t, *J *=* *7.3 Hz, 3H).

### 1-Butyl-3-(1-methyl-1*H*-indazol-6-yl)urea (5b-2).

*n*-Butylisocyanate (25.2 µl, 0.224 mmol) was added to a solution of 4a (30 mg, 0.204 mmol) in pyridine (1 ml), and the reaction mixture was stirred at room temperature for 14 h. After completion of the reaction, the reaction mixture was diluted with ethyl acetate, transferred to a separatory funnel, and washed with water, saturated sodium bicarbonate solution, and brine. The organic layer was dried over anhydrous magnesium sulfate and concentrated *in vacuo*. The crude product was purified by flash chromatography on silica gel (0% to 70% ethyl acetate/hexanes) to obtain the product as a white solid (49.7 mg, 98% yield) as follows: ^1^H NMR (500 MHz, CDCl_3_) δ 7.88 (s, 1H), 7.78 (s, 1H), 7.56 (dd, *J *=* *4.9, 7.9 Hz, 1H), 6.64 to 6.92 (m, 2H), 4.71 to 5.10 (m, 1H), 3.99 (d, *J *=* *4.3 Hz, 3H), 3.22 to 3.33 (m, 2H), 1.44 to 1.56 (m, 2H), 1.29 to 1.41 (m, 2H), 0.83 to 0.99 (m, 3H).

### *N*-(1-Methyl-1*H*-indazol-6-yl)pentanamide (5c-2).

Triethylamine (20 µl, 0.142 mmol) was added to a solution of 4a (19 mg, 0.13 mmol) in dichloromethane (1 ml). To this mixture, pentanoyl chloride (17 µl, 0.142 mmol) was added dropwise at 0°C. The reaction mixture was allowed to warm to room temperature and was then stirred for 16 h. After completion of the reaction, the reaction mixture was diluted with ethyl acetate and transferred to a separatory funnel. The organic layer was washed with saturated aqueous sodium bicarbonate solution followed by water and brine. The organic layer was dried over anhydrous magnesium sulfate, filtered, and concentrated *in vacuo*. The crude product was purified by flash chromatography on a silica gel (gradient, 0% to 70% ethyl acetate/hexanes) to obtain the product as a white solid (22.5 mg, 75% yield) as follows: ^1^H NMR (500 MHz, d_6_-acetone) δ 9.26 (br s, 1H), 8.24 (s, 1H), 7.84 (s, 1H), 7.61 (d, *J *=* *8.5 Hz, 1H), 7.08 (dd, *J *=* *1.7, 8.7 Hz, 1H), 4.00 (s, 3H), 2.41 (t, *J *=* *7.48 Hz, 2H), 1.68 (quin, *J *=* *7.55 Hz, 2H), 1.40 (qd, *J *=* *7.4, 14.9 Hz, 2H), 0.93 (t, *J *=* *7.5 Hz, 3H).

The same basic procedure was performed for the synthesis of *N*-(1-methyl-1*H*-indazol-6-yl)propionamide (5c-1) as follows: ^1^H NMR (500 MHz, CDCl_3_) δ 8.19 (br s, 1H), 7.88 (s, 1H), 7.78 (m, 1H), 7.57 (d, *J *=* *8.54 Hz, 1H), 6.83 (d, *J *=* *8.54 Hz, 1H), 3.99 (s, 3H), 2.44 (q, *J *=* *7.32 Hz, 2H), 1.26 (t, *J *=* *7.48 Hz, 3H).

### Ethyl (1-methyl-1*H*-indazol-6-yl)carbamate (5d-1).

Triethylamine (36.8 µl, 0.264 mmol) was added to a solution of 4a (35.6 mg, 0.241 mmol) in dichloromethane (2 ml). Ethyl chloroformate (25.3 µl, 0.264 mmol) was then added dropwise at 0°C, and the reaction mixture was allowed to warm to room temperature. After 16 h, the reaction mixture was diluted with ethyl acetate and transferred to a separatory funnel. The organic layer was washed with saturated aqueous sodium bicarbonate solution followed by water and brine. The organic layer was dried over anhydrous magnesium sulfate, filtered, and concentrated *in vacuo*. The crude product was purified by flash chromatography on silica gel (0% to 70% ethyl acetate/hexanes) to obtain the product as a white solid (28.3 mg, 54% yield) as follows: ^1^H NMR (500 MHz, CDCl_3_) δ 7.89 (s, 2), 7.59 (d, *J *=* *8.5 Hz, 1H), 6.73 to 6.83 (m, 2H), 4.27 (q, *J *=* *7.0 Hz, 2H), 4.03 (s, 3H), 1.34 (t, *J *=* *7.2 Hz, 3H).

### Butyl (1-methyl-1*H*-indazol-6-yl)carbamate (5d-3).

A solution of 4a (12.5 mg, 0.084 mmol) and 1,1'-carbonyldiimidazole (15.0 mg, 0.093 mmol) in dichloromethane (1 ml) was stirred under reflux conditions for 4 h. After the starting material was consumed, *n-*butanol (1 ml) was added and the reaction mixture was refluxed for an additional 12 h. After completion of the reaction, the reaction mixture was concentrated *in vacuo*. The crude product was purified by flash chromatography on a silica gel (gradient, 0% to 70% ethyl acetate/hexanes) to afford the product as a white solid (16.5 mg, 79% yield) as follows: ^1^H NMR (500 MHz, CDCl_3_) δ 7.82 (s, 1H), 7.53 (d, *J *=* *8.54 Hz, 1H), 6.65 to 6.74 (m, 2H), 4.14 (t, *J *=* *6.56 Hz, 2H), 3.96 (s, 3H), 1.50 to 1.68 (m, 2 H + H from H_2_O), 1.38 (qd, *J *=* *7.40, 15.03 Hz, 2H), 0.90 (t, *J *=* *7.32 Hz, 3H).

The same basic procedure was followed for the synthesis of *n-*propyl (1-methyl-1*H*-indazol-6-yl)carbamate (5d-2) (24.2 mg, 76% yield) as follows: ^1^H NMR (500 MHz, CDCl_3_) δ 7.89 (s, 1H), 7.59 (d, *J *=* *8.54 Hz, 1H), 6.78 (dd, *J *=* *1.53, 8.54 Hz, 2H), 4.17 (t, *J *=* *6.71 Hz, 2H), 4.03 (s, 3H), 1.73 (sxt, *J *=* *7.14 Hz, 2H), 1.00 (t, *J *=* *7.32 Hz, 3H).

### *N*-Methyl-*N*-(1-methyl-1*H*-indazol-6-yl)butane-1-sulfonamide (5e).

NaH (47.2 mg, 1.18 mmol) was added to a vigorously stirred, ice-cold solution of *N*-(1-methyl-1*H*-indazol-6-yl)butane-1-sulfonamide (5a-1) (78.8 mg, 0.295 mmol) in dimethylformamide (3 ml) in four portions. The reaction mixture was maintained at 0°C for 30 min and then warmed to room temperature. Iodomethane (73.5 µl, 1.18 mmol) was added dropwise to the reaction mixture, and the reaction mixture was stirred for 16 h. The reaction mixture was quenched with water and diluted with ethyl acetate. The reaction mixture was transferred to a separatory funnel, washed with water three times, and dried over anhydrous magnesium sulfate. The reaction mixture was concentrated on a rotary evaporator, and the residue was purified by flash chromatography on silica using ethyl acetate as the eluent to afford the product as a yellow solid (70 mg, 85% yield) as follows: ^1^H NMR (500 MHz, CDCl_3_) δ 7.97 (s, 1H), 7.72 (d, *J *=* *8.5 Hz, 1H), 7.48 (s, 1H), 7.13 (d, *J *=* *8.5 Hz, 1H), 4.07 (s, 3H), 3.42 (s, 3H), 2.96 to 3.06 (m, 2H), 1.82 (m, 2H), 1.42 (m, 2H), 0.92 (t, *J *=* *7.32 Hz, 3H).

### 1-Methyl-*N*-pentyl-1*H*-indazol-6-amine (5f).

Lithium aluminum hydride (0.12 ml of a 1 M solution in tetrahydrofuran, 0.12 mmol) was added dropwise to a solution of 5c (14.4 mg, 0.06 mmol) in tetrahydrofuran (0.5 ml) at 0°C. The reaction mixture was allowed to warm to room temperature and was then stirred for 16 h. After completion of the reaction, the reaction mixture was diluted with ethyl acetate and transferred to a separatory funnel. The organic layer was washed with saturated aqueous sodium bicarbonate solution followed by water and brine. The organic layer was dried over anhydrous magnesium sulfate, filtered, and concentrated. The crude product was purified by flash chromatography on silica gel (0% to 70% ethyl acetate/hexanes) to obtain the product as a white solid (12.4 mg, 95% yield) as follows: ^1^H NMR (500 MHz, CDCl_3_) δ 7.76 (s, 1H), 7.43 (d, *J *=* *8.54 Hz, 1H), 6.48 (dd, *J *=* *1.83, 8.85 Hz, 1H), 6.30 (s, 1H), 3.95 (s, 3H), 3.17 (t, *J *=* *7.17 Hz, 2H), 1.69 (quin, *J *=* *7.17 Hz, 2H), 1.31 to 1.49 (m, 4H), 0.94 (t, *J *=* *7.02 Hz, 3H).

### *N*-(3-Methyl-1*H*-indazol-5-yl)butane-1-sulfonamide (5g).

*n*-Butyl sulfonyl chloride (29 µl, 0.22 mmol) was added to a solution of 4 h (30 mg, 0.20 mmol) in pyridine (1 ml), and the reaction mixture was stirred at room temperature for 16 h. After completion of the reaction, the reaction mixture was diluted with ethyl acetate and transferred to a separatory funnel. The organic layer was washed with saturated aqueous sodium bicarbonate solution followed by water and brine. The organic layer was dried over anhydrous magnesium sulfate, filtered, and concentrated. The crude product was purified by flash chromatography on a silica gel (gradient, 30% to 70% ethyl acetate/hexanes) to obtain the product as a white solid (36.8 mg, 69% yield) as follows: ^1^H NMR (500 MHz, CDCl_3_) δ 7.60 (d, *J *=* *1.53 Hz, 1H), 7.43 (d, *J *=* *8.85 Hz, 1H), 7.28 (d, *J *=* *1.83 Hz, 1H), 6.55 (s, 1H), 3.03 to 3.09 (m, 2H), 2.59 (s, 3H), 1.78 to 1.89 (m, 2H), 1.43 (sxt, *J *=* *7.45 Hz, 2H), 0.91 (t, *J *=* *7.32 Hz, 3H).

### Accession number(s).

Atomic coordinates and structure factors for KasA, KasA-DG167, KasA-5a-30, and KasA-5g have been deposited in the Protein Data Bank, and assigned PDB codes 5W2O, 5W2P, 5W2Q, and 5W2S, respectively.
