# Supplementary figures and images for: Synergistic Lethality of a Binary Inhibitor of Mycobacterium tuberculosis KasA
Source: mBio. 2018 Dec 18;9(6):e02101-17. doi: 10.1128/mBio.02101-17 (PMC6299220; doi:10.1128/mBio.02101-17)

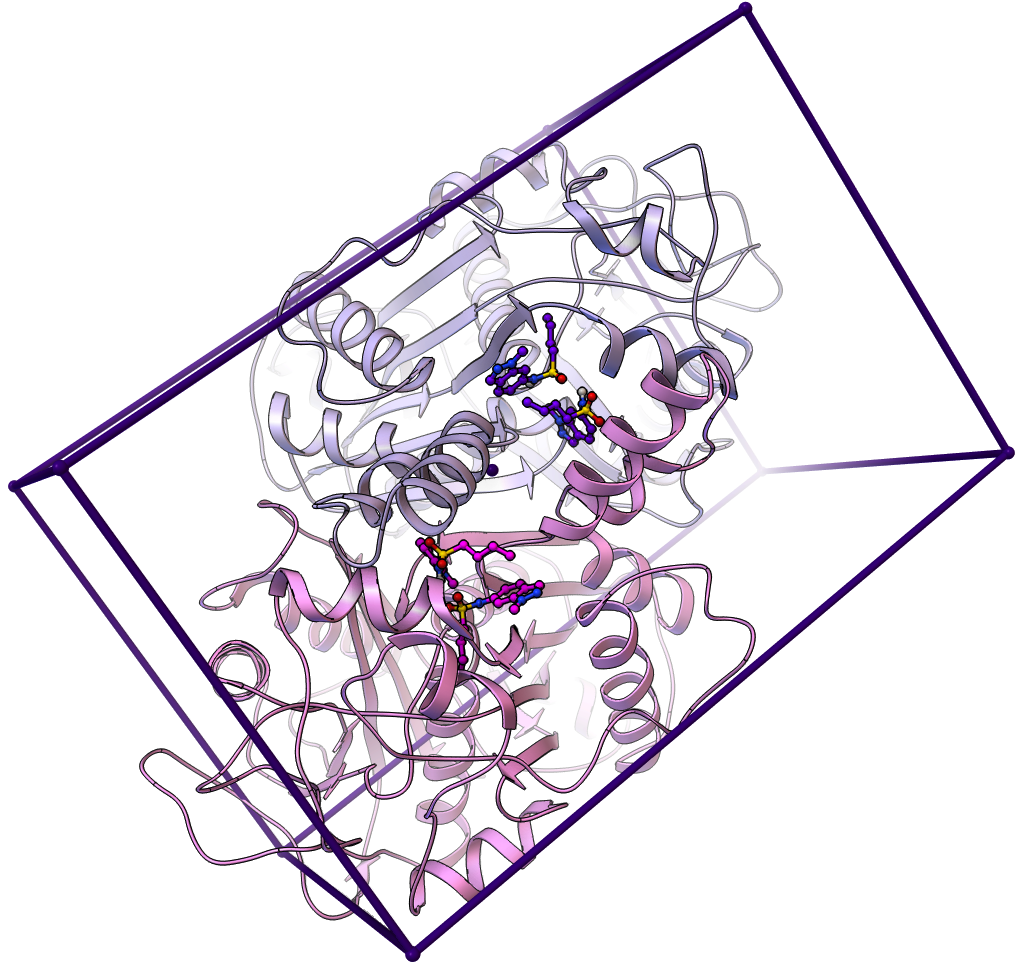

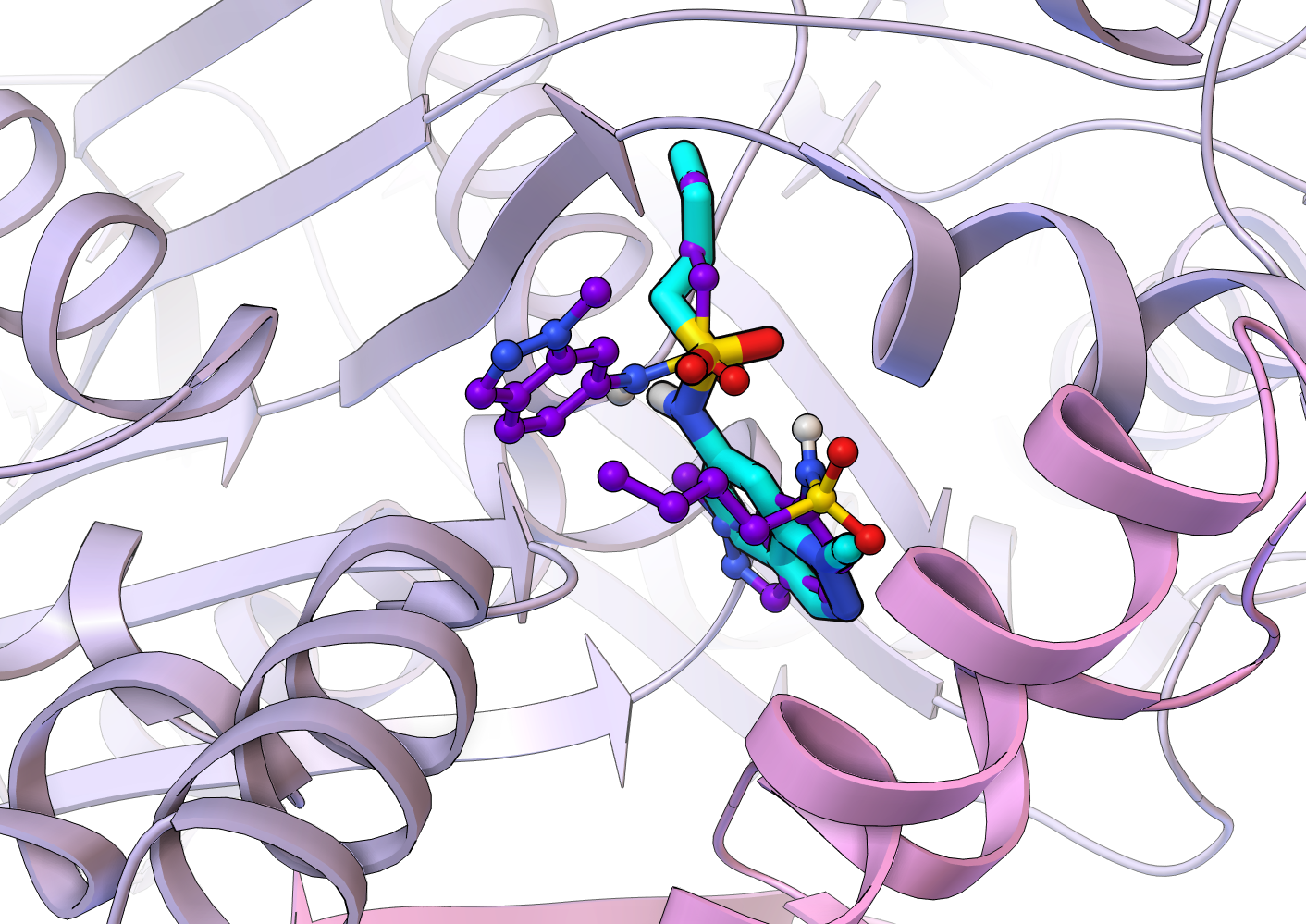


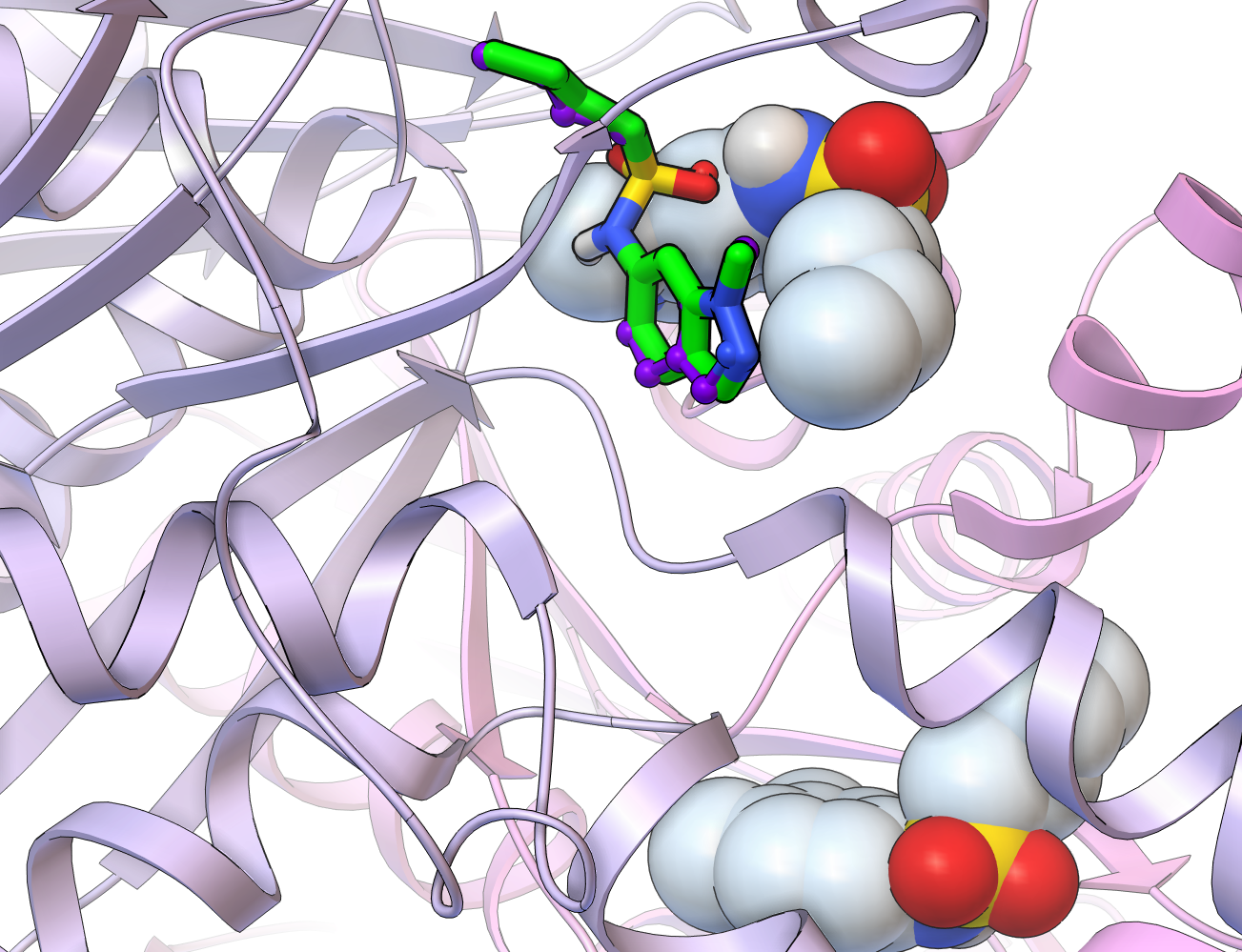

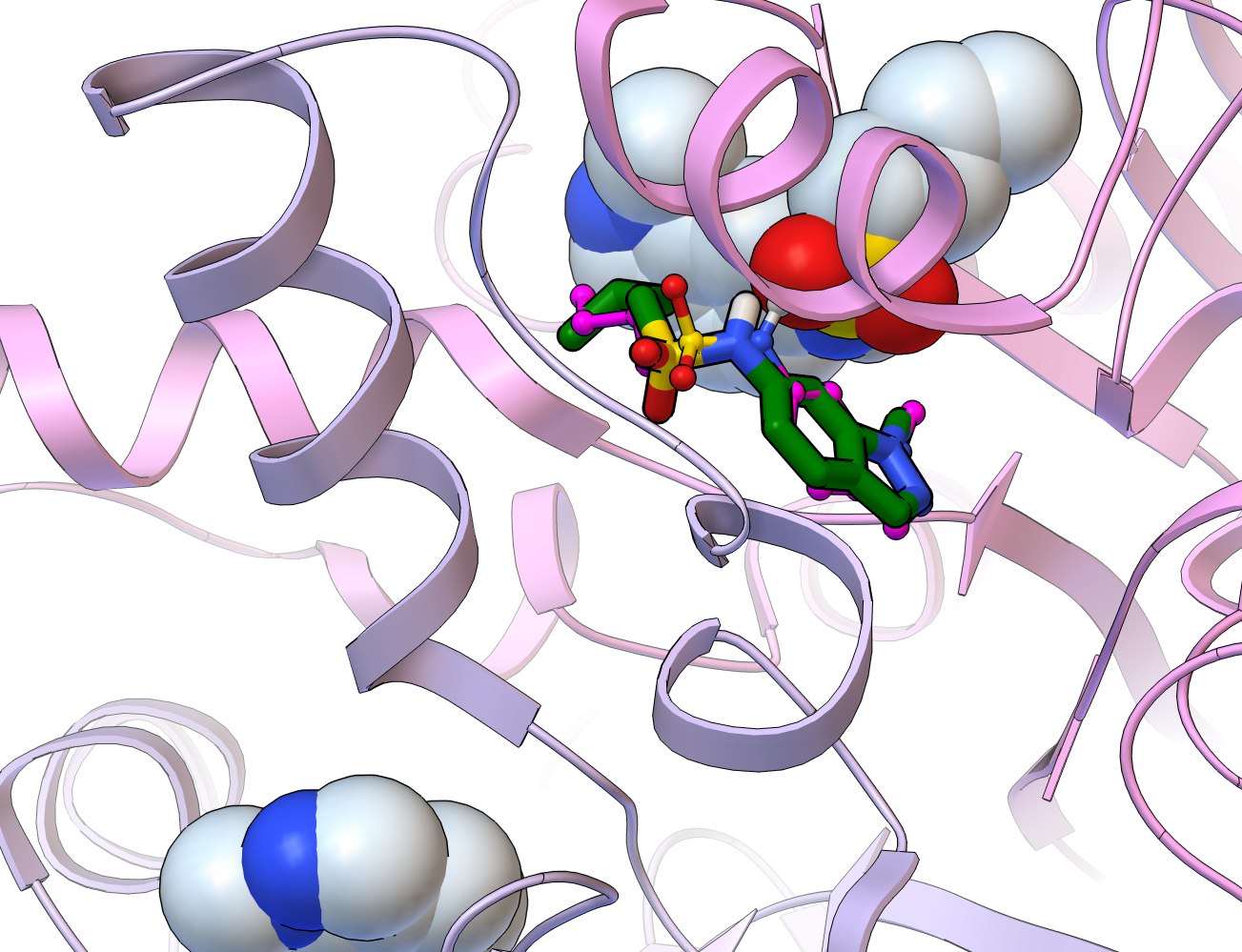


B

A

D

C

**Fig. S1**.

Supplement: FIG S1 [file mbo006184230sf2.docx]

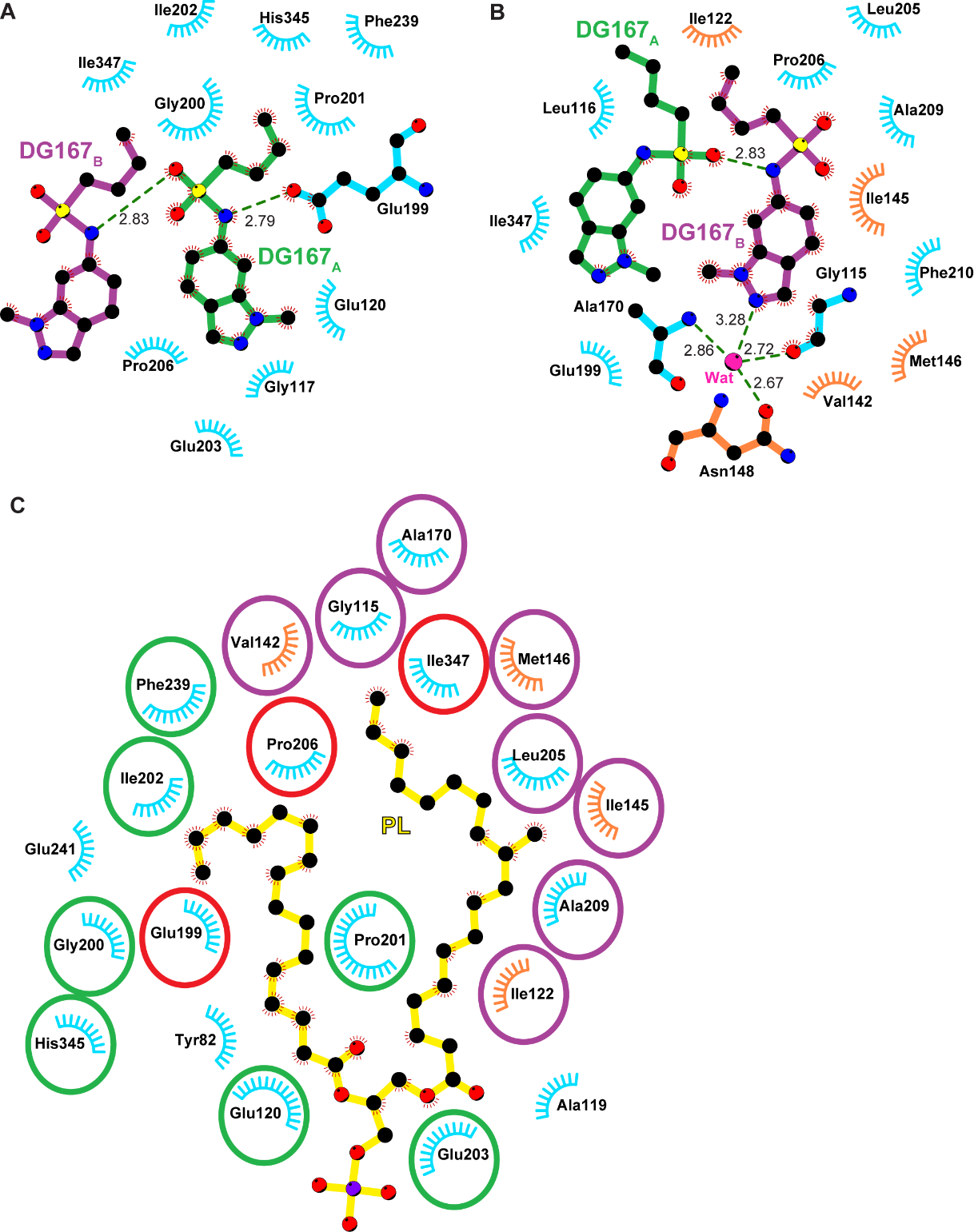


**Fig. S2**.

Supplement: FIG S2 [file mbo006184230sf10.docx]

**
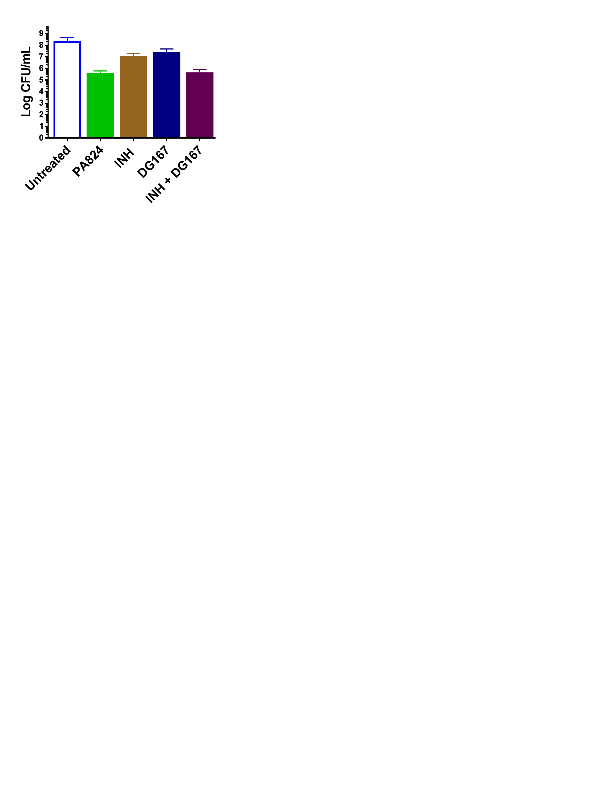
**

**B)**

**A)**

**D)**

**C)**

**Fig. S4.**

Supplement: FIG S4 [file mbo006184230sf4.docx]

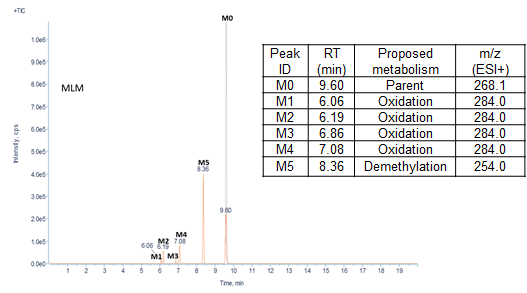


**A)**

**
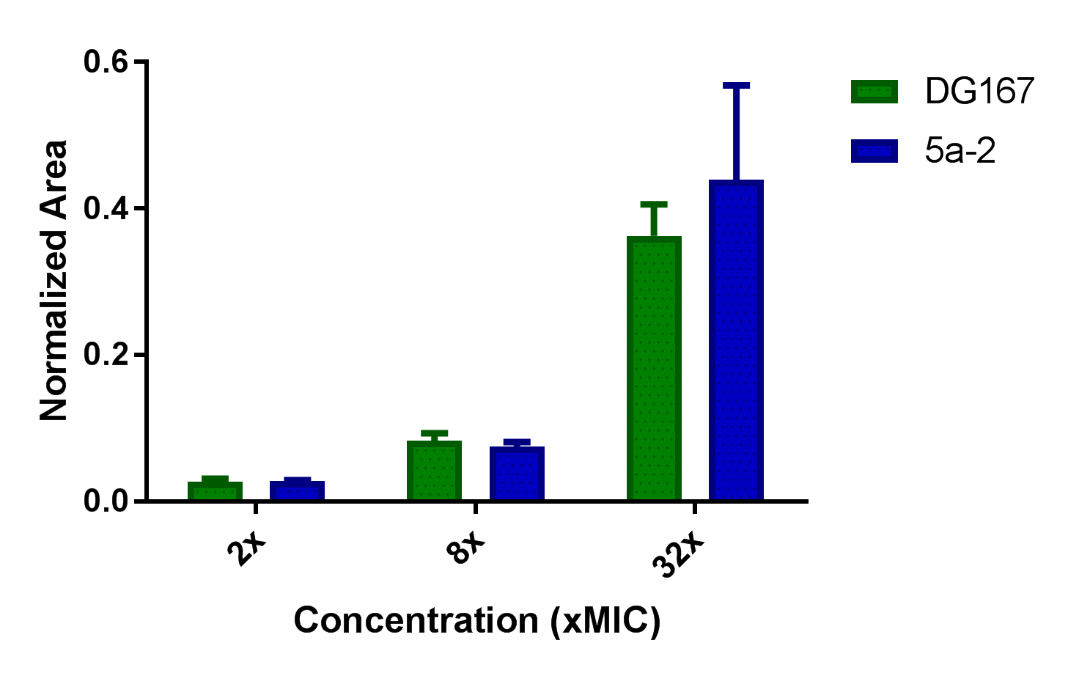
**

**B)**

**Fig. S5.**

Supplement: FIG S5 [file mbo006184230sf5.docx]

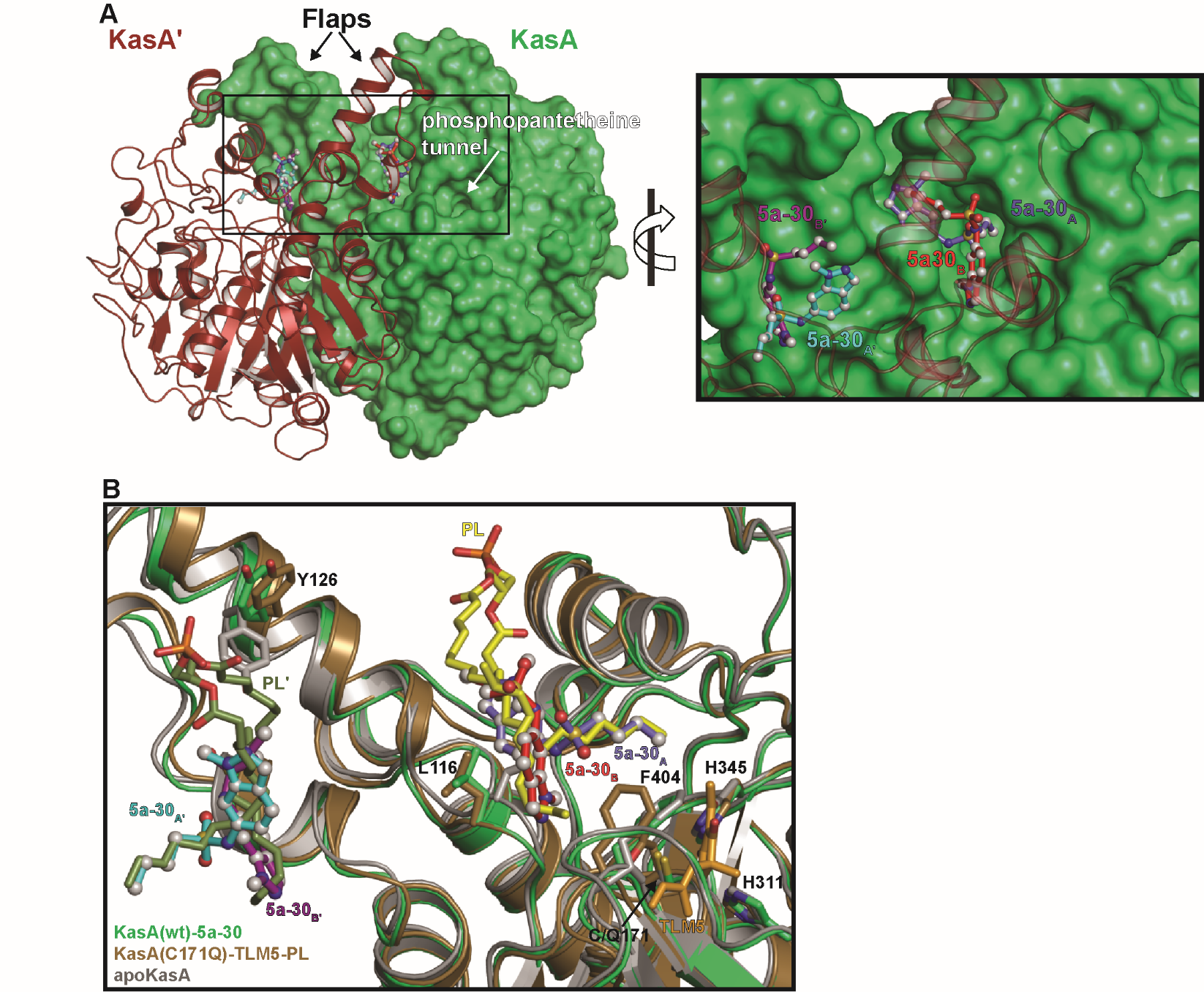


**Fig. S6**.

Supplement: FIG S6 [file mbo006184230sf6.docx]

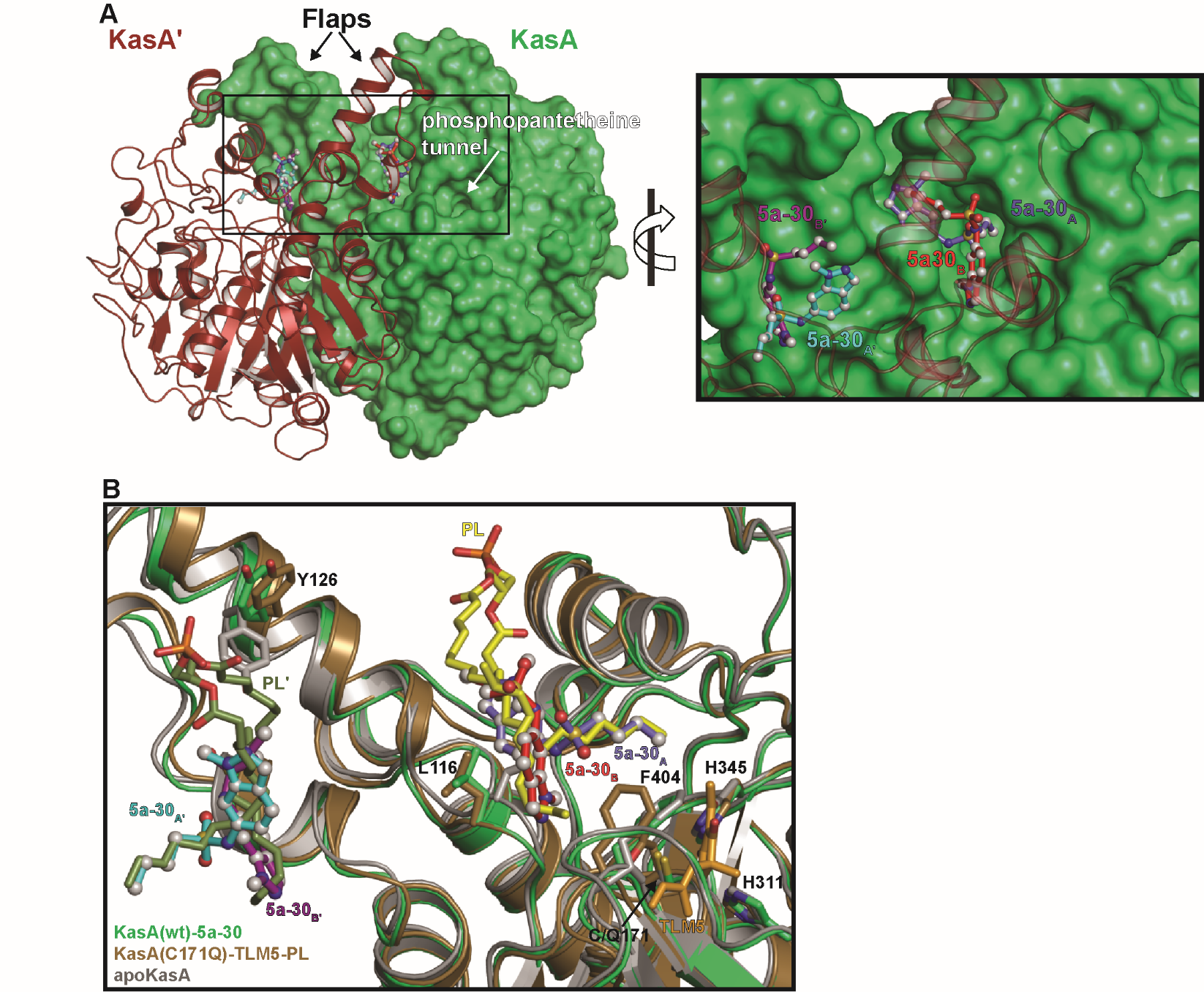


**Fig. S7**.

Supplement: FIG S7 [file mbo006184230sf7.docx]
