# Supplementary material for: Synergistic Lethality of a Binary Inhibitor of Mycobacterium tuberculosis KasA
Source: mBio. 2018 Dec 18;9(6):e02101-17. doi: 10.1128/mBio.02101-17 (PMC6299220; doi:10.1128/mBio.02101-17)
Supplement: TABLE S1 [file mbo006184230st1.docx]

**Table S1.** Data collection and refinement statistics.

|  | KasA | KasA-DG167 | KasA-**5a-30** | KasA-**5g** |
| --- | --- | --- | --- | --- |
| **Data collection** |  |  |  |  |
| Space group | P3_1_21 | P3_1_21 | P3_1_21 | P3_1_21 |
| Cell dimensions | | | | |
| a, b, c (Å) | 77.39, 77.39, 147.01 | 77.30, 77.30, 145.21 | 77.29, 77.29, 145.13 | 77.04, 77.04, 145.81 |
| α, β, γ (°) | 90.0, 90.0, 120.0 | 90.0, 90.0, 120.0 | 90.0, 90.0, 120.0 | 90.0, 90.0, 120.0 |
| Resolution (Å) | 50.00-1.80 (1.83-1.80) | 50.00-2.00 (2.03-2.00) | 50.00-1.80 (1.83-1.80) | 50.00-2.40 (2.46-2.40) |
| Wavelength (Å) | 1.1808 | 1.1808 | 1.1808 | 0.88557 |
| Completeness (%) | 99.9 (100.0) | 99.0 (99.3) | 100.0 (100.0) | 99.9 (99.4) |
| R_sym_ (%) | 7.1 (67.3) | 4.6 (62.7) | 5.0 (84.7) | 7.0 (58.8) |
| CC^1/2^ | 0.999 (0.872) | 0.999 (0.764) | 1.000 (0.756) | 0.999 (0.944) |
| Average I / σI | 27.5 (3.5) | 36.7 (3.6) | 38.5 (2.3) | 31.5 (3.7) |
| Redundancy | 6.69 | 6.29 | 7.28 | 9.67 |
| Total reflections | 321,338 | 219,551 | 344,325 | 195,916 |
| Unique reflections | 48,010 | 34,892 | 47,310 | 20,269 |
| **Refinement** | | | | |
| R_work_ / R_free_ (%) | 15.38 (20.24) / 17.95 (23.11) | 15.73 (19.34) / 18.62 (22.81) | 15.58 (22.70) / 18.62 (29.93) | 18.41 (22.75) / 21.37 (26.08) |
| **Number of atoms** | | | | |
| All atoms | 3386 | 3268 | 3312 | 3161 |
| Protein | 3060 | 3034 | 3041 | 3047 |
| DG167 | - | 36 | - | - |
| 5a-30 | - | - | 36 | - |
| 5g | - | - | - | 18 |
| Glycerol | 24 | 6 | 6 | 12 |
| TCEP | 16 | 16 | 16 | - |
| 2-propanol | 12 | 12 | 12 | 4 |
| Ions | 2 | 1 | 1 | 1 |
| Water | 272 | 163 | 200 | 79 |
| **Average B-factor (Å^2^)** | | | | |
| All atoms | 28.10 | 41.74 | 37.11 | 58.64 |
| Protein | 26.96 | 41.34 | 36.41 | 58.90 |
| Ligands | 52.43 | 57.00 | 54.85 | 57.73 |
| Water | 36.01 | 42.70 | 41.46 | 48.88 |
| **R.m.s. deviations** | | | | |
| Bond lengths (Å) | 0.008 | 0.007 | 0.006 | 0.003 |
| Bond angles (°) | 0.868 | 0.821 | 0.832 | 0.552 |
| **Ramachandran statistics** | | | | |
| Favored (%) | 96.18 | 97.82 | 97.60 | 97.09 |
| Allowed (%) | 2.39 | 1.70 | 2.16 | 2.67 |
| Outliers (%) | 0.48 | 0.48 | 0.24 | 0.24 |

Data collection and refinement statistics. R_sym_ = Σ_h_ Σ_i_ | I_i_(h) - <I(h)> | / Σ_h_ Σ_i_ I_i_(h), where I_i_(h) is the i^th^ measurement of h and <I(h)> is the mean of all measurements of I(h) for reflection h. R_work_ = Σ ||F_o_| - |F_c_|| / Σ |F_o_|, calculated with a working set of reflections. R_free_ is R_work_ calculated with only the test set of reflections. Data for the highest resolution shell are given in parentheses. The structures were determined using single crystals.
