# Supplementary material for: Synergistic Lethality of a Binary Inhibitor of Mycobacterium tuberculosis KasA
Source: mBio. 2018 Dec 18;9(6):e02101-17. doi: 10.1128/mBio.02101-17 (PMC6299220; doi:10.1128/mBio.02101-17)
Supplement: FIG S3 [file mbo006184230sf3.docx]

**Fig. S3**.
