# Supplementary material for: Synergistic Lethality of a Binary Inhibitor of Mycobacterium tuberculosis KasA
Source: mBio. 2018 Dec 18;9(6):e02101-17. doi: 10.1128/mBio.02101-17 (PMC6299220; doi:10.1128/mBio.02101-17)
Supplement: TABLE S3 [file mbo006184230st8.docx]

**Table S3.** Profile of DG167

| **Parameter** | **value** |
| --- | --- |
| *M. tuberculosis* MIC (μM) | 0.39 |
| Vero Cell CC_50_ (μM) | 23 |
| Vero cell-based SI | 59 |
| J774 Cell CC_50_ (μM) | 94 |
| Kinetic solubility in pH 7.4 PBS (μM) | 324 |
| MLM stability t_1/2_ (min) with/without NADPH | 10.1 / >300 |
| MLM stability Cl_int_ (μL/min/mg protein) | 68.9 |
| Caco-2 Permeability P_A-B_ / P_B-A_ (x10^-6^ cm/s) | 71.8 / 45.6 |
| Chemical stability t_1/2_  (min) at  pH 2  pH 7  pH 9 | 11500  Stable  3470 |
| Human plasma protein binding (%) | 91.9 |
| Human plasma stability at 5 h (%) | 109 |
| Human recovery (%) | 92.0 |
| Mouse plasma protein binding (%) | 79.0 |
| Mouse plasma stability @ 5 h (%) | 99.1 |
| Mouse recovery % | 90.7 |
| Cytochrome P450 IC_50_ (μM)  1A2  2C9  2C19  2D6  3A4 | >50  >50  12.4  >50  >50 |
| hERG IC_50_ (µM) | >20 |
| PK in mice 25 mg/kg PO dose  AUC[0-t] h*ng/mL  Bioavailability (%) | 8083.96  92.3 |
| PK in mice 5 mg/kg IV dose  AUC[0-t] h*ng/mL  t_1/2_ (h) | 1751.25  0.33 |

.
